# Quantifying Synergy: A Systematic Review of Mixture Toxicity Studies within Environmental Toxicology

**DOI:** 10.1371/journal.pone.0096580

**Published:** 2014-05-02

**Authors:** Nina Cedergreen

**Affiliations:** Department of Plant and Environmental Sciences, University of Copenhagen, Frederiksberg, Denmark; CSIR-Central Drug Research Institute, India

## Abstract

Cocktail effects and synergistic interactions of chemicals in mixtures are an area of great concern to both the public and regulatory authorities. The main concern is whether some chemicals can enhance the effect of other chemicals, so that they jointly exert a larger effect than predicted. This phenomenon is called synergy. Here we present a review of the scientific literature on three main groups of environmentally relevant chemical toxicants: pesticides, metal ions and antifouling compounds. The aim of the review is to determine 1) the frequency of synergy, 2) the extent of synergy, 3) whether any particular groups or classes of chemicals tend to induce synergy, and 4) which physiological mechanisms might be responsible for this synergy. Synergy is here defined as mixtures with minimum two-fold difference between observed and predicted effect concentrations using Concentration Addition (CA) as a reference model and including both lethal and sub-lethal endpoints. The results showed that synergy occurred in 7%, 3% and 26% of the 194, 21 and 136 binary pesticide, metal and antifoulants mixtures included in the data compilation on frequency. The difference between observed and predicted effect concentrations was rarely more than 10-fold. For pesticides, synergistic mixtures included cholinesterase inhibitors or azole fungicides in 95% of 69 described cases. Both groups of pesticides are known to interfere with metabolic degradation of other xenobiotics. For the four synergistic metal and 47 synergistic antifoulant mixtures the pattern in terms of chemical groups inducing synergy was less clear. Hypotheses in terms of mechanisms governing these interactions are discussed. It was concluded that true synergistic interactions between chemicals are rare and often occur at high concentrations. Addressing the cumulative rather than synergistic effect of co-occurring chemicals, using standard models as CA, is therefore regarded as the most important step in the risk assessment of chemical cocktails.

## Introduction

### Background

Cocktail effects and synergistic interactions of chemicals in mixtures are an area of great concern to both the public [1], [2] and regulatory authorities in the US and Europe ([3] and references therein). There are two general aspects underlying this concern: The first is the uncertainty as to whether we are monitoring and regulating the most harmful chemicals? The second concerns whether the chemicals we regulate on a single compound basis, and deem “safe”, potentiate or are being potentiated by other chemicals so that they jointly exert a larger effect than predicted? The latter is called synergy, and is one of the factors that create uncertainty around models proposed for the implementation in chemical risk assessment of mixtures. For those legislations where mixtures are considered, which are few, dose- or concentration additivity is proposed as the default model [3]. But are synergistic interactions really an area that should concern us? Earlier reviews have shown that synergistic interactions, at least within pesticide mixtures and realistic low-dose chemical mixtures in mammals, are a rather rare phenomenon, constituting approximately 5% of the tested mixture combinations [4]–[7]. This percentage is rather low given the fact that experiments are often designed to search for synergistic interactions, thereby biasing the databases towards synergistic interactions. If, however, these 5% are combinations that often co-occur in humans and the environment, they might nonetheless be of quantitative importance. Hence, if we could identify the groups of chemicals that are likely to induce synergistic interactions, special precautions could be taken in the risk assessment of these chemicals. Identifying the potential synergists would reduce the uncertainty of using the models proposed for risk assessment of mixtures of the remaining 95% of antagonistic or non-interacting chemicals [3].

The aim of this review is therefore to define which groups of chemicals are involved in well documented synergistic interactions, and if possible, to identify the mechanisms behind their synergistic effects. This will be done within three large groups of chemicals that often co-occur in the environment at measurable concentrations: The first group consists of pesticides, which is probably the most well studied chemical group within ecotoxicological mixtures studies. This is not only due to the use of chemical mixtures in pesticide formulations and tank mixtures and the resulting co-occurance in agricultural areas, but just as much because of the in depth knowledge of their physiological mode of action. This makes them ideal candidates for testing mixture models based on chemical mode of action and understanding the physiological mechanisms behind possible interactions [8], [9]. The second group of chemicals are metals. Metals typically co-occur in potentially toxic concentration in relation to mining, smelting and other industrial activities and a substantial body of literature on metal mixtures is available [10]. The last group are antifouling biocides, which consist both of traditional organic biocides, organo-metals and metal ions [11], making this group a mixture of the two above thereby opening the possibility of finding other synergistic mechanisms. Antifoulants co-occur in harbour areas and marine and freshwater areas with substantial boat traffic [12], [13].

Chemical mixtures from waste water treatment plants, oil spills, industrial effluents and other sources yielding very complex mixtures have not been included for two reasons: The first is that they are often chemically very poorly described; hence, we do often not know which chemicals cause the majority of the toxicity [14], [15]. The second is that the probability for severe interactions decrease when the number of pollutants adding to the joint toxicity increase [16], [17], hence, severe interactions are more likely to occur when a few chemicals dominate the overall toxicity, as is more often seen for e.g. pesticide pollution, in comparison to effluent pollution [18].

### The Definition of Synergy

Defining synergy as two or more chemicals exerting a larger effect than predicted implies that we can predict joint effects of chemicals under certain assumptions. The aim of being able to do so, has been a research topic for more than a century [19], and the two major concepts underlying all valid assessments of joint chemical effects were framed already in the first part of the twentieth century by Loewe and Muischnek (1926) and Bliss (1939), respectively [20], [21]. Loewe and Muischnek (1926) based their concept on the assumption that all chemicals in a mixture acted on the same biological target site and therefore could be viewed as being dilutions of each other, each having a different chemical potency. Hence, if the chemical potency of chemical A and B in a binary mixture was based on the Effect Concentration (*EC*) of each chemical causing *x*% effect on any endpoint in a test-system (1/*EC_xA_* and 1/*EC_xB_*), then the sum of the concentration of chemicals (*c_A_* and *c_B_*) multiplied with their respective potency in a mixture provoking x% effect would be equal to 1 [20]:
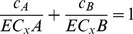
(1)


The concept has been re-invented several times since 1926 and has received many names such as Loewe Additivity, Dose Addition, The Additive Dose Model or Concentration Addition, depending on whether it has been used within pharmaceutical, agricultural, toxicological or ecotoxicological sciences [22]. In this review we will use the term Concentration Addition (CA). Bliss (1939) worked with test-systems where mortality was the endpoint, and added another way of looking at mixtures, in the cases where the tested chemicals obviously did not affect the organisms through a similar molecular target. Different target sites were by Bliss defined by their concentration-response curves having different shapes [21]. Bliss viewed death by a chemical as a stochastic event. The probability of surviving or dying due to exposure to several chemicals acting on independent targets in the organism could therefore be calculated based on probabilities of surviving or dying from exposure to the individual chemicals [21]. Hence, the probability of surviving two independently acting chemicals (R_mix_) would be equal to the probability of surviving the first chemical (R_1_) multiplied by the probability of surviving the second chemical (R_2_). Or, if assessing the probability of dying from two independently acting chemicals (E_mix_), this is equal to the probability of dying from the first chemical (E_1_) plus the probability of dying from the second chemical (E_2_), minus the probability of dying from both chemicals (E_1_×E_2_) [21].
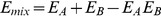
(2)


This concept has likewise been re-invented several times and has been named Bliss Independence, Response Multiplication, Response Addition, Effect Addition, Independent Action a.o. depending on the inventor and context [22]. In this review we will use the term Independent Action (IA). Both concepts can be extended to an infinite number of chemicals and can be used to predict mixture toxicity effects of all mixture ratios and effect levels, providing that entire dose- or concentration response relationships for the single chemicals in the desired test-system are available. Often such data are not available and reduced approaches must be used. A recent review of mixture models and their uses can be found in Cedergreen et al. (2013) [22]. How they are proposed to be used in different chemical legislation is reviewed by Backhaus and Faust (2010) [3]. Common for both concepts is also the assumption that the chemicals do not interact chemically or affect the toxicity of each other [20], [21]. If the chemicals do interact, the joint effects might deviate from the predictions resulting either in the before mentioned synergistic effects or in antagonistic effects, which are defined as smaller effects than predicted [22].

Synergy can therefore be defined in relation to two basic concepts: CA and IA. Empirical evidence, however, shows that even mixture toxicity of dissimilarly acting compounds can be described with a high level of accuracy with CA, as well as with IA, despite their different underlying assumptions [6], [23]. CA generally generate slightly more conservative predictions (predicting larger effects than IA), and as databases on chemicals often only provides *EC_x_* data or No Observable Effect Concentrations (NOECs) or Lowest Observable Effect Concentrations (LOECs) which only makes CA predictions possible and not IA, CA is most often the recommended model for risk assessment purposes [3]. In this review, synergy is therefore defined in relation to CA predictions.

Experimental data are always determined with variance. For mixture studies this applies both to the toxicity data of the individual compounds used to make the model prediction, and to the tested mixture toxicity data. The consequence of this is that small deviations from the reference models can be difficult to detect statistically and repeat experimentally [24]. Biologically significant and reproducible synergy is therefore here defined as a more than two fold deviation from CA, as was also proposed by Belden et al (2007) [5]. That is, the concentration predicted to yield a certain effect is more than twice the concentration actually observed giving the proposed effect [5]. Belden calls the ratio of predicted versus observed effect concentrations for the Model Deviation Ratio (MDR) [5]. Many of the mixtures showing MDRs slightly below two, most likely also include true synergists. But to exclude false positives and to focus on combinations where the size of the synergistic interactions might be of quantitative importance, we have chosen to set the MDR limit defining synergy at two.

## Materials and Methods

### Identification of Experiments

**Figure 1:**
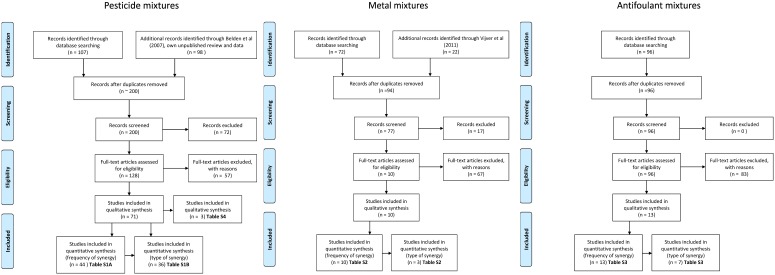
PRISMA 2009 Flow Diagram [90]. A flow diagram depicting the process of selection of records used in the review for the three main groups of toxicants: Pesticides, metals and antifoulants. Data selection has, for pesticides and metals, been built on previous reviews and data compilations, given in the top right text-box, supplemented with database searched using ISI Web of Science. Search criteria and criteria for selecting eligible records are given in the Material and Methods section. For each toxicant the search resulted in two types of databases: One to determine the frequency of synergy in a randomly selected number of mixtures studies, and another focussing only on defined synergistic mixtures. It should be noted that many records contain data on several independent mixtures studies; hence the number of records given in the figure does not match the number of selected studies reported in the results section. References to tables in supporting material giving the raw data on specific chemical mixtures, test species, endpoint and timecourse of the experiment, and the record providing the information are given in the figure.

A flow chart of the record selection for each of the three toxicant groups is presented in Figure 1. To evaluate the frequency of chemicals, chemical mixtures and species groups involved in synergistic (MDR>2), additive (0.5≤MDR≤2) and antagonistic (MDR<0.5) mixture experiments, the database of Belden et al. (2007) was used for the pesticides, the one of Vijver et al (2011) was used as a starting point for the metals, while our own data-collection was used for the antifoulants (Figure 1, Supporting information: Table 1A, 2 and 3).

For the pesticide mixtures, Belden et al (2007 and our own database on synergistic interactions was expanded with more recent studies screening the database ISI Web of Science using the search words “pesticide*”, “mixture*” and “synerg*” in the period 2008–2013. For the metals, the review by Vijver et al (2011) [10] was supplemented by newer studies using ISI Web of Science and the search words “metal*”, “mixture*”, “synerg*” and “toxic*” for the period 2009–2013. The antifoulant mixture compilation using ISI Web of Science and the search words “antifoul*” and “mixture*” for the time period 1990–2013 to be able to detect the frequency of synergy in a similar way as had been done in the study by Belden et al. (2007).

Only studies complying with the criteria developed by Belden et al (2007) were used: Mixture studies should be conducted using only pure substances. Hence, studies using formulated pesticide or formulated antifouling biocides were excluded, as the formulation products could affect the results. Studies using metals in the form of nano-particles were likewise excluded. To avoid biasing the database with similar experiments, duplicated experiments using the same mixture and species presented in the same manuscript were entered in the database as one study, but giving the MDRs of each individual replicate. If multiple mixture ratios were tested in the same experiment, the MDR from the mixture ratio closest to the ratio where both chemicals contributed equally to the toxicity (equipotent ratio) was used in the cases of isobole designs, where several mixture ratios were tested. Otherwise the numerically larger MDR was used. Finally, the experiment had to be conducted in a way that an MDR could be calculated. That is, comparable *ECx* values or Toxic Units (*1/EC_x_*) from individual compounds and their mixtures should be available either directly or from reading off graphs. From each study, the following information was collected: The chemicals involved, the species tested, the higher taxonomic group of the species, monitored endpoint and duration of the toxicity test, and the original reference where the raw data were reported. Studies on species communities were not included.

It should be noted that the published data does not represent a random selection of chemical mixtures tested on representative ecological species, but rather represent mixtures selected because of co-occurrence or suspicion of synergy tested on standard laboratory species. The choice of chemicals biases the database towards detecting synergies, while the choice of robust laboratory organisms, on the other hand, might give conservative estimates on synergies as they might not represent the most susceptible species.

All data treatments were done in excel.

## Results

### The Frequency of Synergy

Figure 1 presents the selection process of record for the study. Several of the records reported more than one mixture toxicity experiment. In the following the individual mixture toxicity experiments will be discussed. The records from where data has been retrieved can be found in the tables S1–S4 in File S1 in the supporting information. A PRISMA Checklist for reviews is given in Checklist S1.

#### Pesticide mixtures

The database of Belden et al (2007) provided data on 207 pesticide mixtures of which 194 were binary and another 13 consisted of more than two pesticides [5].

#### Metal mixtures

Evaluating the meta-analysis of Vijver et al (2011) on metal mixtures according to the criteria set by Belden et al (2007), reduced the number of usable studies from 22 to 6 studies reporting 10 experiments where MDR could be calculated and another 7 experiments, where data shown on graphs could be evaluated as being over or under-predicted by CA (Table S2 in File S1). Of the remaining 15 studies, five studies only allowed IA predictions and 10 studies reported metal tissue accumulations, but not effects. Since there is not always a straight forward correlation between tissue accumulation and toxic effect [25], [26] these were disregarded. A recent paper of Xu et al (2011) [27] added another 11 metal mixtures where MDR-values could be calculated, making it a total of 28 mixtures from 8 studies tested on 7 species. Of these, 21 mixtures were binary while the remaining 7 mixtures consisted of more than two metals.

#### Antifoulants mixtures

**Figure 2:**
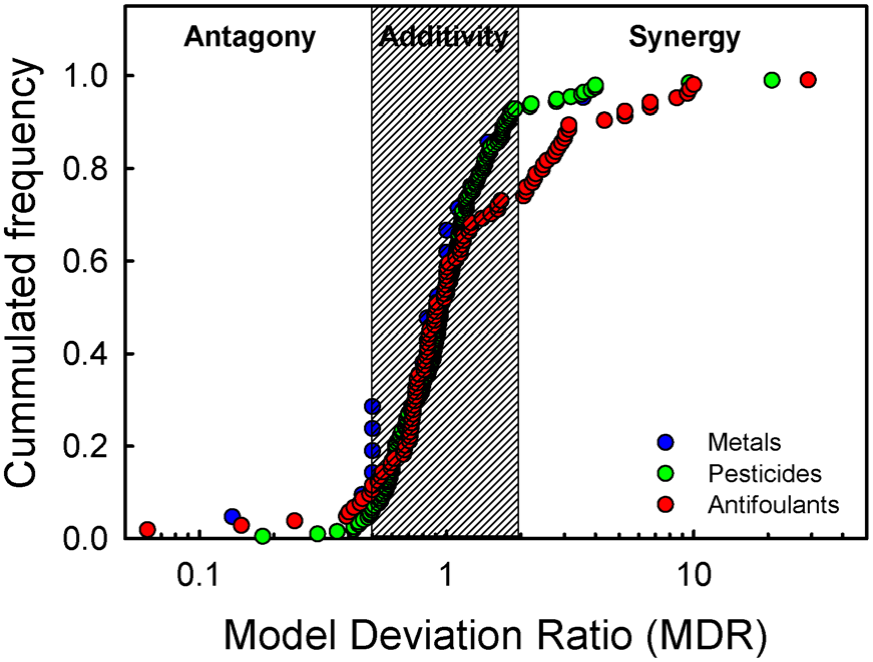
Cummulated frequency of Model Deviation Ratios. Cummulated frequency of Model Deviation Ratios. (MDR) of binary mixtures of pesticides (*n* = 195), metals (*n* = 20), and antifoulants (*n* = 103). The hatched interval where 0.5≤MDR≤2 defines the mixtures that deviates less than two-fold from a Concentration Addition predictions. Mixtures having MDR values<0.5 are termed antagonistic, while mixtures with MDR values>2 are synergistic.

For antifoulants 136 mixtures where MDR-values could be calculated were found. These were presented in 14 studies comprising mixtures of 20 chemicals tested on 15 different species (Table S3 in File S1). There were 103 binary mixtures and 33 mixtures with more than two chemicals. The frequencies of synergy in the binary mixtures were 7%, 3% and 26% for pesticides, metals and antifoulants, respectively, while 88%, 86% and 64% was within two fold of the CA prediction (Figure 2). For the 13 pesticide mixtures where more than two chemicals were included, only one was synergistic [5], while for the 33 antifoulant mixtures with more than two chemicals 61% showed severe synergy (Table S3 in File S1).

### Types of Synergy

#### Pesticide mixtures

**Figure 3:**
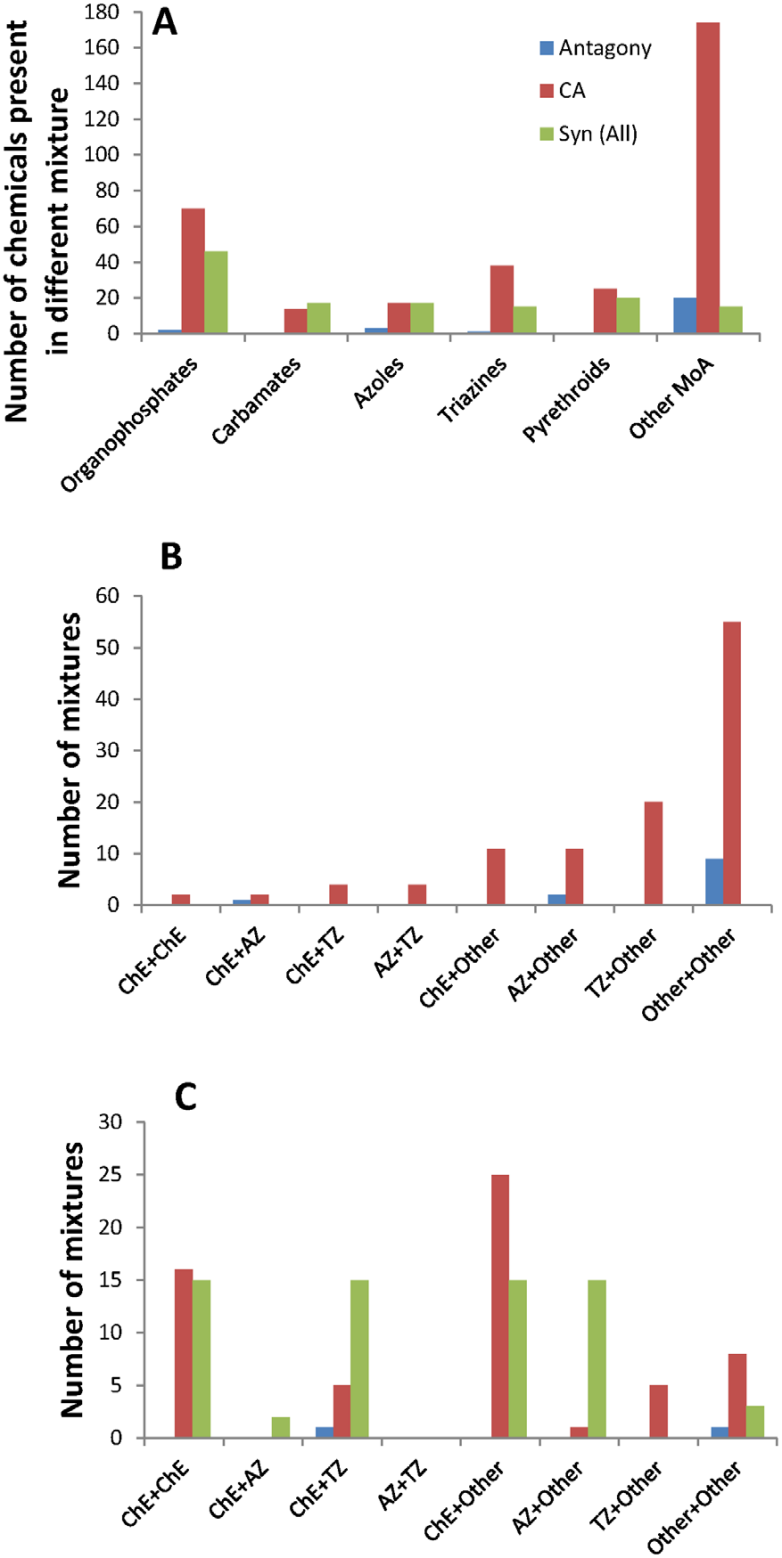
Frequency of pesticide antagony, additivity and synergy. Figure 2A shows the number of times a pesticide belonging to the group of organophosphates, carbamates, azoles, triazines, pyrethroids or some other Mode of Action (other MoA) occur in a binary mixture resulting in antagony (blue bars), concentration additivity (CA) (red bars) or synergy (green bars). In figure B and C, the number of binary combinations of cholinesterase inhibitors (ChE) (The organophosphates and carbamates), azoles (AZ), triazines (TZ) and other Modes of Action (Other) resulting in either antagony, concentration additivity or synergy are shown for mixtures tested on B) auto-tropic organisms (plants and algae, *n* = 120) or C) heterotrophic organisms (microorganisms and animals, *n* = 128).

In addition to Belden et al (2007) [5] and the review by Cedergreen et al (2008) [28] another 84 papers were reviewed for synergy where the MDR ratios were >2. This resulted in a database on synergistic interactions including 73 cases of synergy from both Belden et al (2007) and the data search compiled from 36 studies. These studies tested the effect of combinations of 54 pesticides on 27 different species. Of all the mixture combinations, 69 were binary mixtures while the remaining four mixtures consisted of combinations of three or five organophosphate insecticides or eight chloroacetamide herbicide safeners (Table S1B in File S1). Dividing the pesticides into groups with common modes of action according to Tomlin (2002) [29] showed that particularly five groups of pesticides were overrepresented in the synergistic mixtures. These were the organophosphate and carbamate insecticides (Cholinesterase inhibitors), azole fungicides (Ergosterol biosynthesis inhibitors), triazine herbicides (Photosystem II inhibitors) and pyrethroid insecticides (interferes with sodium channels in nerve cells) (Figure 3A). Grouping the cholinesterase inhibitors together and looking at which of the binary combinations of the above pesticide groups induced synergy in auto-trophic organisms (plants and algae) and hetero-trophic organisms (microorganisms and animals) showed no cases of synergy within the autotrophic organisms (Figure 3B). In the group of hetero-trophic organisms 69 of the 73 synergistic mixtures (95%) contained either cholinesterase inhibitors (organophosphates or carbamates) or azole fungicides (Figure 3C). The remaining four mixtures were the before mentioned mixture of 8 herbicide safeners, a mixture of a pyrethroid with an organochloride insecticide, a pyrethroid insecticide and a piperidine fungicide and a photosystem II (PSII) inhibiting herbicide and a growth regulator (Table S1B in File S1). Of the 69 binary mixtures 76% contained a cholinesterase inhibitor and another 24% an azole fungicide (Figure 3C). The triazines only entered in synergistic mixtures together with either chlorpyriphos, diazinon, malathion, methidathion, methyl-parathion, which belong to the phosphorothioate and phosphorodithioates class of organophosphates, or trichlorfon, a phosphate class organophosphate. Pyrethroids, on the other hand, only entered in synergistic mixtures together with azole fungicides.

An evaluation of which types of the pesticides from the review of Belden et al (2007) were dominant in the antagonistic mixtures and those conforming to CA, showed that cholinesterase inhibitors and azole fungicides made up 29% of the antagonistic mixtures and 48% of the mixtures conforming to CA (Figure 3B and C), which is considerably less than the 95% of the synergistic mixtures. Hence, though these modes of action were present in all types of mixtures, they were clearly overrepresented in the mixtures displaying synergistic interactions. The triazines occurred in 1% of the antagonistic mixtures, 22% of the concentration additive mixtures and in 12% of the synergistic mixtures. Hence, triazines did not seem to occur particularly frequently in the synergistic mixtures, and when they did, only in mixtures with the before mentioned organophosphates. The 19 triazine mixtures with an MDR<1 were dominated by Auxin transport inhibitors, branched chain- and aromatic amino acid synthesis inhibitors, while the 19 triazine mixtures with MDR values between 1 and 2 were dominated by organophosphates, PSII inhibitors and cell division inhibiting herbicides. All 22 additive mixtures including pyrethroids, were mixtures with organophosphates, carbamates or other pyrethroids (Table S1A in File S1).

#### Metal mixtures

Going through the 55 selected potential papers found on ISI Web-of-Science using the key-words given above only revealed two additional studies with three experiments where MDR>2 could be estimated from figures (Table S2 in File S1). Hence, despite the large numbers of studies made on metal mixtures, well documented severe synergistic metal-metal interactions seem to be rare. The four binary mixtures giving synergy were, Cd+Zn, Cu+Zn, Cu+Cd and Cd+As tested on the shrimp *Penaeus setiferus*, the fish *Gobiocypris rarus* and the water-flee *Daphnia magna* (Table S2 in File S1).

#### Antifoulants mixtures

**Figure 4:**
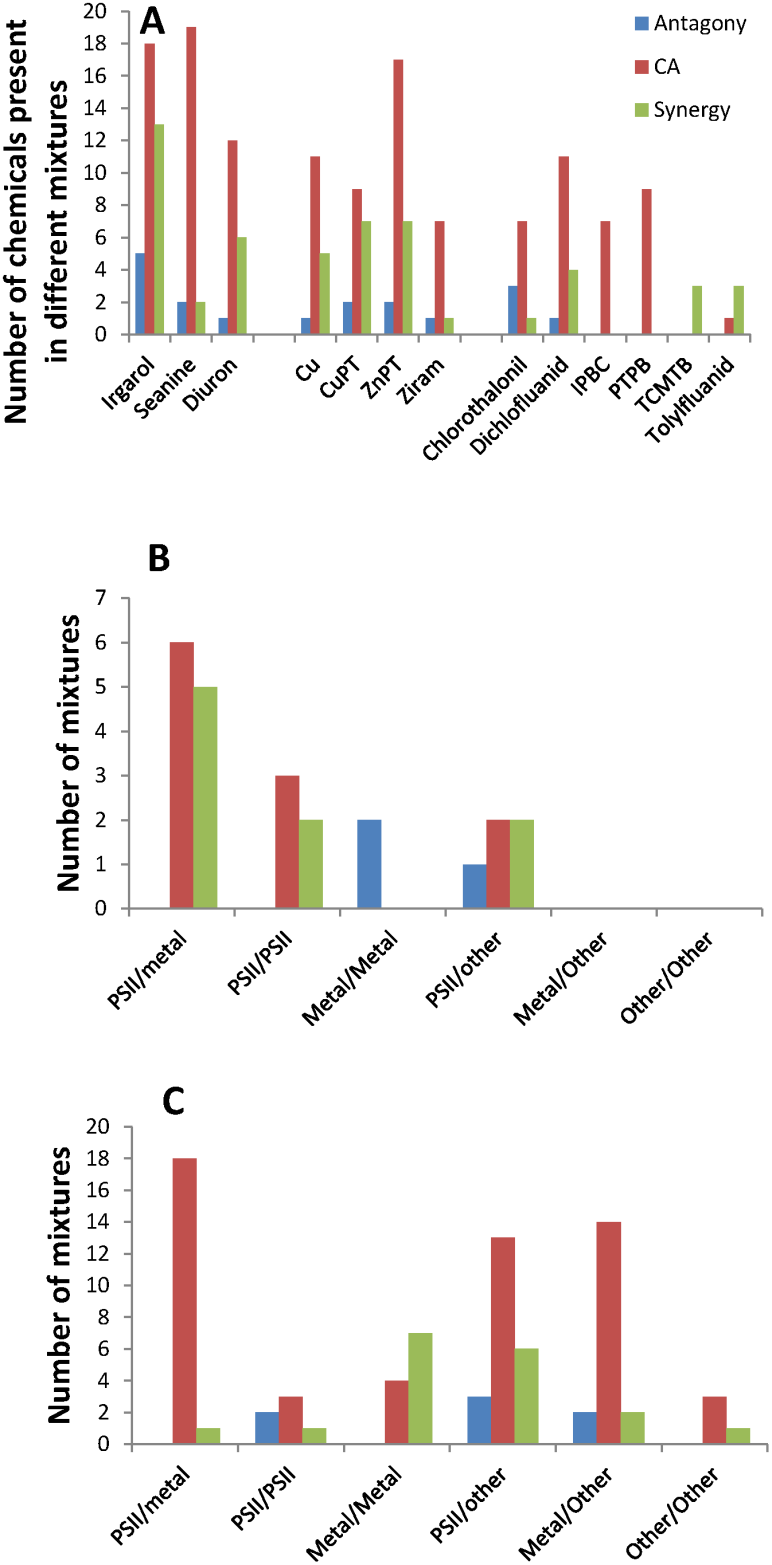
Frequency of antifoulant antagony, additivity and synergy. Figure 3A shows the number of times each of the antifoulants occur in a binary mixture resulting in antagony (blue bars), concentration additivity (CA) (red bars) or synergy (green bars). Antifoulants occurring in less than 1% of the mixtures were excluded. In figure B and C, the number of binary combinations of photosystem II herbicides (PSII) metal ions or metal containing compounds (Metal) and other organic compounds (Other) resulting in either antagony, concentration additivity or synergy are shown for mixtures tested on B) auto-tropic organisms (plants and algae, *n* = 23) or C) heterotrophic organisms (microorganisms and animals, *n* = 80).

In the antifoulants database (Table S3 in File S1), we found 47 cases of synergy from 8 studies, testing the effect of mixtures of 12 chemicals on 9 organisms. Another 7 chemicals were tested that did not occur in any of the synergistic mixtures. The antifoulants were more difficult to categorise according to physiological mode of action compared to the pesticides, as this information is not required for registration. While pesticides are often developed to act physiologically very specifically in specific target organisms, antifoulants are selected to be toxic to the wide range of organisms settling on ship hulls. Hence, their physiological mode of action is more likely to be general, targeting physiological pathways important for a broad range of species. Hence, the analysis of the frequency of chemicals in synergistic, additive and antagonistic mixtures were done on the individual chemicals roughly divided into three groups: Herbicides (2,4-D, atrazine, irgarol 1051, Seanine 211, and diuron), metals and metal containing organic compounds (Cd, Cu, Cu Pyrethrione (PT), Zn, ZnPT, Ziram and Tributhyltin (TBT)), and other organic compounds (chlorothalonil, dichlorfluanid, 3-iodo-2-propynyl butylcarbamate (IPBC), pyridine triphenylboron (PTPB), 2-thio cyano methyl thio benzothiazole (TCMTB) and tolylfluanid). Chemical class and proposed modes of action are given in Table 1. For the 103 binary mixtures the frequency of synergy was markedly higher than the frequency of antagony for mixtures containing either irgarol or diuron, Cu, CuPT or ZnPT, TCMTB, dichlorofluanid or tolyfluanid (Mixtures including Cd, Zn, or TBT were excluded in this analysis as they were included in <3 binary mixtures each) (Figure 4A). Analysing the frequency of binary mixtures combined of the above defined three overall groups for the 23 binary studies on plants and algae and the 80 studies on animals and microorganisms separately, showed that all synergistic mixtures tested on plants or algae contained a PSII inhibiting herbicide either in combination with another PSII inhibiting herbicide, or metal or an organic antifoulant (Figure 4B). This is contrary to the pesticide study, where no synergy was found in studies on auto-trophic organisms at all (Figure 3B and C, Table S1B in File S1). For the autotrophic organisms the PSII inhibitors were present in slightly more additive than synergistic mixtures (Figure 4B). For the group of heterothrophs, PSII inhibiting herbicides also caused synergy, particularly together with dichlorfluanid, tolyfluanid and TCMTB (Figure 4C, Table S3 in File S1). Combinations of two metal containing compounds induced synergy in seven of 11 cases for this group (64%). Hence, contrary to mixtures of metal ions (Table S2 in File S1), mixtures of organically bound metals seem to be much more potent in inducing synergy.

**Table 1:** The overall group, name and proposed Modes of Action (MoA) of the antifouling compounds.

Group	Name	IUPAC name	Mode of Action
Photosystem IIinhibitors	Atrazin	6-chloro-*N* ^2^-ethyl-*N* ^4^-isopropyl-1,3,5-triazine-2,4-diamine	Inhibits the electron transport inphotosystem II
	Irgarol1051	2-tert-butylamino)-4-(cyclopropylamino)-6-(methylthio)-1,3,5-triazine	Inhibits the electron transport inphotosystem II
	Seanine211	4,5-dichloro-2-n-octyl-4-isothiazoline-3-one	Inhibits the electron transport inphotosystem II
	Diuron	3-(3,4.dichlorophenyl)-1,1-dimethylurea	Inhibits the electron transport inphotosystem II
Metals andorganometals	Cd	Cadmium ion	General toxicant, interacts with enzymes^a^
	Cu	Copper ion	General toxicant, interacts withenzymes^a^
	CuPT	Copper 2-pyridinethiol-1-oxide	General toxicant, interacts withenzymes^a^
	Zn	Zink ion	General toxicant, interacts withenzymes^a^
	ZnPT	Zinc 2-pyridinethiol-oxide	General toxicant, interacts withenzymes^a^
	Ziram	Zinc bis(N,N’-dimethyl)-dithiocarbamate	Dimethyldithiocarbamate fungicide with Zn. Inhibitor of enzymes containing copper ions or sulfhydryl groups, including P450monooxygenases of the CYP 2A6 group^b^
	TBT	tri-butyl-tin-chloride	PSII inhibitor (with tin), endocrine disruptor^c^
Fungicides	Chlorothalonil	Tetrachloroisophthalonitrile	Conjugation with, and depletion of, thiols (particularly glutathione) fromgerminating fungal cells, leading todisruption of glycolysis andenergy production, fungistasis andfungicidal action.
	Dichlofluanid	*N*-dichlorofluoromethylthio-*N’,N’*-dimethyl-*N*-phenylsulfamide	Multi-site mode of action, non-specific thiol reactant, inhibiting respiration.
	IPBC	3-iodo-2-propynyl butylcarbamate	AChE inhibitor and fungicide and bactericide^d^
	PTPB	Pyridine triphenylboron	Fungicide^d^
	TCMTB	2-thio cyano methyl thio benzothiazole	Fungicide, Inhibitor of mitochondrial electron transport^c^
	Tolylfluanid	*N*-dichlorofluoromethylthio-*N*’,*N*’-dimethyl-*N*-*p*-tolylsulfamide	Multi-site mode of action, non-specific thiol reactant, inhibiting respiration.

a Altenburger, 2011 [88].

b Walker, 2009 [49].

c Fernandez-Alba et al, 2002 [89].

d Zhou et al, 2006 [40].

Particularly for the fungicides, which have multiple and often undefined modes of action, different target sites are given in different references. For herbicides and fungicides used as pesticides we use the definition of Tomlin 2002 [29]. For the remaining compounds, the source of the MoA are given as footnotes.

**Figure 5:**
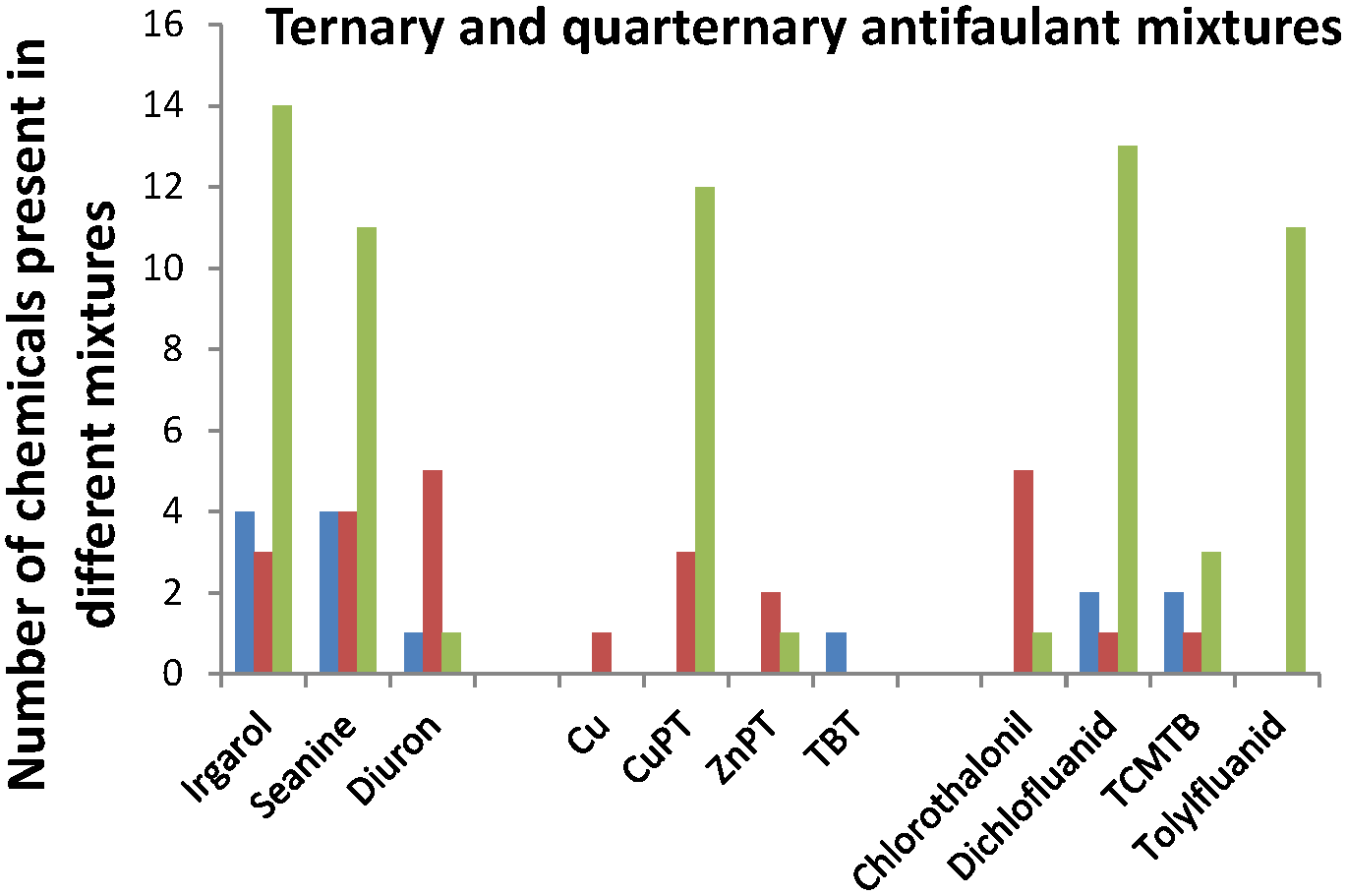
Frequency of antifoulant interactions in ternary and quaternary mixtures. The number of times each of the antifoulants occur in a ternary (*n* = 23) or quaternary (*n* = 10) mixture resulting in antagony (blue bars), concentration additivity (CA) (red bars) or synergy (green bars).

Of the 23 ternary mixtures of antifoulants and the ten quarternary mixtures, four mixtures were antagonistic, nine additive and the remaining 20 mixtures had an MDR>2 (Table S2 in File S1). The frequency of occurrence of the different antifoulants in antagonistic, additive and synergistic mixtures is shown in figure 5, confirming that particularly irgarol, Seanine, CuPT, dichlofluanid and tolylfluanid often occur in synergistic mixtures.

#### Synergistic mixtures of metals and organic compounds

In the search for synergistic mixtures, some mixtures showed up that could not be categorised as either pesticides or metals, as they contained both. Synergistic interactions between metals and pesticides seemed to be quite frequent compared to synergistic mixtures of metals alone, but since a comprehensive database on mixture experiments of metals and pesticides has not been made, this cannot be tested. Table S4 in File S1 in the Supplementary material show 11 mixtures of metals and pesticides from three studies, of which eight had a MDR value >2.

## Discussion

### Which are the Chemicals Causing Synergy?

The review showed that for pesticides, the combinations causing synergy were not random but included either cholinesterase inhibitors or azole fungicides in 95% of the described cases. The proposed mechanisms behind these synergies are relatively well investigated, as discussed below. The synergy frequency for metal ion mixtures was very low, hence no general conclusion in terms of which compounds caused synergy could be made. When metal ion synergy occurred, it was in the mg L^−1^ concentration range for three of the four cases [30], [31]. These concentrations are high, compared to the concentrations normally found in metal polluted waters being in the lower ng to µg L^−1^ range [32], [33]. For the antifouling compounds synergistic interactions were also related to specific chemical groups, though more synergistic combinations of different chemical groups were involved than seen for the pesticides (Figure 3, 4). The high frequency of synergistic interactions observed for the antifoulants, particularly in the mixtures with more than two active ingredients, is most likely due to the selection for compounds able to induce synergy in antifouling products, which most often are composed of more than one active ingredient [34]. The mechanisms behind the synergistic interactions of the antifoulants are, contrary to what is seen for the pesticides, rarely investigated. In the following, the proposed mechanisms behind the synergistic interactions of pesticide, metal ions and antifouling mixtures are discussed.

### Mechanisms Causing Synergistic Interactions

Interactions between chemicals can basically affect six processes that are important for the resultant toxicity of a chemical towards an organism: bioavailability, uptake, internal transportation, metabolization, binding at the target site and excretion. The synergistic interactions identified in the present study are most likely caused by interactions around one or more of these processes. In the following, known mechanisms behind the identified synergistic interactions will be discussed in terms of which processes are most likely affected by the interactions.

#### Bioavailability

Interactions between chemicals can take place outside the organism, with one chemical affecting the availability of the other. This is commonly seen for metal ions, where ion speciation and competition for binding sites to organic matter in soil, sediments and the water phase can change free ion availability and composition [35]–[37]. If a less toxic ion replaces a bound or chelated ion with a higher toxicity, this will lead to apparent synergistic interactions, if the toxicities are estimated based on total metal concentrations rather than bioavailable concentrations. These types of interactions, however, most often occur when binding sites are limited. Hence, either the metal ion concentrations are high, or the binding site density low, as would for example be the case with ions in water with low concentrations of dissolved organic matter or mineral ions as calcium carbonate and other salts [37]. None of the four metal-metal ion synergies found in this study (Table S4 in File S1) allow for an assessment of whether the synergistic interactions occurred due to changed ion availability, as only total metal concentrations were given. Changes in speciation outside the organism as a cause of synergistic interaction has, however, been well documented for mixtures of pyrithione antifoulants [38]. When ZnPT and Cu ions are mixed together the more toxic CuPT complex is formed, making the mixture more toxic than predicted from the toxicities of ZnPT and Cu alone [38]–[40]. As the affinity of pyrithione for Cu is higher than for Zn, then the equilibrium between the metal-pyrithione complexes and free pyrithione will be shifted in favour of CuPT [41]. If there is a metal ion surplus in the ZnPT and CuPT synergistic mixture observed by Koutsaftis and Aoyama (2006) [42], a shift towards a larger proportion of CuPT might be taking place. It could therefore be hypothesised that changes in speciation outside the organism is a main mechanism behind all the reported synergistic interactions of metal/metal mixtures among the antifoulants (Figure 4C), apart from the mixture of Cu and Ziram [40].

#### Uptake rates and transport to the target site

One chemical can affect the uptake rate of the other by for example competition at biological ligands or competitive inhibition of transport proteins, as is often observed for interactions on metal uptake ([35], [36] and references herein); though not all studies explicitly describe external ion availabilities, making it difficult to determine whether the interactions measured on internal concentration stem from interactions on bioavailability or on uptake.

Interactions on uptake rates have, however, also been measured for combinations of organic contaminants. When Belden and Lydy (2000) investigated the synergistic interactions between the herbicide atrazine and the organophosphate insecticide chlorpyriphos, they found that the addition of atrazine increased chlorpyriphos uptake by 40% [43]. This increase in contaminant uptake was proposed to be caused by an increased oxygen consumption, leading to higher ventilation rates and thereby higher uptake rates of a contaminant as chlorpyriphos, which is predominantly taken up over the gills. Increased ventilation alone could not explain the observed four-fold increase in toxicity. Hence, though it is likely that many contaminants will increase ventilation rates when the organisms start spending energy metabolizing them, thereby increasing uptake of other contaminants taken up over gills, lungs and tracheid’s, the quantitative importance of this extra uptake is most likely of little importance for the more severe synergistic cases reported in the literature.

Potential important effects on uptake was also proposed by Kennaugh et al (1993) in a study on the effect of the known synergist Piperonyl Butoxide (PBO) on the cytochrome P450 mediated metabolic rate of the pyrethroid insecticide Permethrin in permethrin resistant and wildtype *Helicoverpa armigera* [44]. The ability of PBO to break the 20-fold resistance could not be explained by differences in P450 monooxygenase mediated permethrin detoxification rates, since they were identical for the resistant and non-resistant genotypes. Hence, it was proposed that PBO instead affected a P450 mediated “penetration resistance” developed by the resistant strain, making the resistant strain take up less pyrethroid. The proposed effect on uptake rates was, however, never confirmed by actual studies of Permethrin uptake. Hence, the P450 mediated “penetration resistance” is still a hypothesis.

Many of the synergists known to enhance uptake belong to the large group of surfactants and other additives added to formulated pesticides with the exact purpose of enhancing the uptake of the active compounds [45]. As this review has excluded all studies with formulated compounds and surfactants, the database does not include examples on surfactant synergies, despite of their frequent use. Though there is a proven effect of the surfactants on uptake of active compounds when hitting their target at high concentrations, it is likely that most lose their “uptake enhancing” potency when diluted in environmental matrices, even though they might still act as dilute pollutants adding to the overall toxicity according to concentration addition. This is supported by a toxicity study on formulated versus technical herbicides on aquatic plants and algae showing no difference in potency for nine out of ten herbicides [46].

The transport rate of one chemical towards its molecular target can be affected by the presence of another chemical, as is for example the proposed mechanisms behind the strong antagonistic responses often seen in plants when a rapidly acting photosynthetic inhibitor is mixed with a slower acting systemic herbicide [47]. No studies have, to my knowledge, shown that one chemical can actively increase the transport of another chemical to their target.Thist is, nonetheless, the proposed mechanism behind many hypotheses regarding nano-particle facilitated increase in chemical toxicity [48], which we will not touch upon here, and therefore cannot be excluded either for chemical/chemical interactions.

#### Metabolic enzyme activities

Alternations of metabolic activity that are the most well investigated mechanisms behind observed synergistic patterns. A chemical can either increase or decrease the metabolization rate of another chemical. Decreased metabolization will typically lead to a higher toxicity than expected, when the toxic effect is caused by the unchanged parent compound. In contrast, increased metabolization will increase the toxicity of chemicals which are metabolically activated.

Synergistic interactions involving azole fungicides are most likely all examples of cases where the metabolization of the pesticides is inhibited by the azole. Azole fungicides are known inhibitors of a wide range of P450 monooxygenases, which are enzymes responsible for the phase I metabolization of lipophilic compounds [49], together with a range of biosynthesis processes in both plants and animals [50], [51]. Hence, the toxicity of lipophilic insecticides such as pyrethroids are often severely enhanced when mixed with azole fungicides [49], [52]–[55].

The synergistic cases involving cholinesterase inhibitors, which made up 76% of all the synergistic pesticide mixtures, most likely all also involve interactions on metabolism. The dominant mechanisms are, however, different depending on which compounds are involved. Basically three mechanisms can be involved:

First, besides the target enzyme acethylcholinesterase (AChE), organophosphate and carbamate insecticides can also inhibit esterases, which are responsible for phase II metabolization of other xenobiotics, including organophosphates and carbamates themselves [49]. Although having the same mode of action and therefore supposedly following concentration addition, mixtures of some organophosphates and carbamates do act synergistically [56]–[61] (Figure 3).

Second, organophosphates, from the phosphorothioate and phosphorodithioates class of organophosphates, must be metabolically activated to their more active oxon form in order to inhibit the target site AChE [43]. This means that compounds that can induce the production of P450 monooxygenases, will increase the rate of oxon formation and hence increase the toxicity of the organophosphates. This mechanism has been proposed as being the main mechanism responsible for the cases of synergy between triazine herbicides and organophosphates [43], [62]. Belden and Lydy (2000) elegantly showed how the amount of polar metabolites of chlorpyriphos increased in *Chironomus tentans* in the presence of atrazine [43], explaining the majority of the observed synergy. Triazine herbicides have also been shown to induce P450 activity in fish [63], [64]. The fact that all the cases of pesticide synergy between triazines and organophosphates include organophosphates belonging to the class of phosphorothioate and phosphorodithioates (chlorpyriphos, diazinon, malathion, methidathion, methyl-parathion) [43], [62], [62], [65]–[68], or being transformed into one [69], [29], indicate that triazine induced P450 induction is the main cause of this synergistic interaction. However, not only triazines induce P450 activity. Many xenobiotics, ranging from polyaromatic hydrocarbons, dioxins and ethanol [49], [70] to natural substances in honey and metal ions [71], [72] are proven P450 inducers. The synergistic interactions between organo(thio-)phosphates and neonicotenoids in the nematode *Caenorhabditis elegans* were also proposed to stem from P450 induction of neonicotenoids [73]. It even seems as if compounds that inhibit P450 activity at high concentrations induce activity at low concentrations or on a longer time-scale. Azole fungicides have, for example, shown to give protective effects against pyrethroid toxicity at low doses in bees [52] and the aquatic invertebrate *Daphnia magna* (pers.obs.), and have been shown to induce synergy together with organophosphates in birds pre-treated with prochloraz [74]–[76]. In the bird studies increased metabolism of the organophosphates was measured, strongly indicating P450 induction [75], [76].

Third and finally, phosphorothioate organophosphates are known to inhibit some types of P450 monooxygenases, thereby not only affecting phase II but also affecting phase I metabolism of xenobiotics [49]. New studies have shown that the inhibitions and activations of different P450 genes are compound specific [77], as are the xenobiotics affinities for the different monooxygenases [49]. Hence, it is likely that the majority of severe synergistic interactions can be explained by interactions on metabolism. Which types of interactions plays the largest role for specific chemical combinations, and at which concentrations and timescales the interactions are most severe for different species, is, however, still largely unexplored.

#### Excretion

As the ability of an organism to excrete a compound is mainly related to its ability to transform xenobiotis to an excretable form, excretion is closely related to metabolization. One exception is active excretion of essential metals as Cu and other ions, for which specific transporters or other excretion systems exist, aiding in keeping internal concentrations within a non-toxic range [78], [79]. It could be hypothesised that interactions on these excretion processes could lead to synergistic interactions if they were in some way inhibited, though none of the synergistic mixtures included in this study have proven these mechanisms to be important.

### Synergistic Interactions where the Mechanisms are Unknown

Apart from the pesticide and metal examples given above, where mechanisms causing synergistic interactions are, if not proven, then at least suggested, the review also revealed synergistic chemical combinations where the mechanisms are unknown. These were mainly the interactions between PSII herbicides with other PSII herbicides, metals or non-azole fungicides in the antifouling mixtures together with the mixtures of metals and organo-metals (Table S3 in File S1) or simply metals and organic pesticides (Table S4 in File S1).

Photosystem II herbicides did not induce synergy in any of the 33 mixtures performed on plants or algae in the pesticide database (Table S1 in File S1). Hence, it was surprising to find that significant synergy was found in nine of the 21 antifoulant mixtures including PSII herbicides when tested on plants or algae. Five of these nine mixtures were with the metals Cd, Cu and Zn, which were not part of any of the PSII mixture in the pesticide database. A proposed synergistic mechanism between metals and PSII inhibitors in autotrophs could be that metals might prevent the repair of not only damaged PSII complexes, which are constantly repaired during photosynthesis [80], but also the damage caused by the reactive oxygen species (ROS) created by the PSII inhibition and the metals themselves, by interacting with enzymes responsible for the repair. The two synergistic PSII/PSII mixtures were between irgarol and diuron, while the remaining two were between irgarol and chorothalonil or TCMTB. The synergies between irgarol and the two general fungicides, chorothalonil and TCMTB, could be similar to the mechanism proposed for the PSII/metal interactions, as both fungicides create ROS [81] and additionally chlorothalonil conjugates with gluthatione [29], an important ROS scavenger. These hypotheses, however, need to be tested.

The mode of action of PSII inhibitors in heterotrophs is largely unknown, as these organisms lack photosystems. The studies on pesticides revealed that triazines, such as irgarol, can induce P450 activity in heterotrophs, thereby enhancing the effect of the organophosphates which needed to be metabolically activated. A study by Suzuki et al (2004) show that also dichlofluanid and chlorothalonil need activation by P450 monooxygenases to reach their full lipid-oxidation potential, with dichlofluanid being far more potent than chlorothalonil [81]. As tolylfluanid is chemically related to dichlofluanid, it might also have to be oxidised by P450 to be fully activated. A study on fish have shown that also the metal ions Cu^+^ and Pb^+^ can induce P450 activity [72]. Five of the seven synergistic mixtures involving PSII inhibitors or metals together with organics are mixtures of the triazine irgarol or Cu and either dichlofluanid or tolylfluanid. In addition, for the twenty synergistic mixtures with more than two antifoulants, these combinations were present in all but three mixtures. It could therefore be hypothesised that the main mechanism behind the synergy between irgarol or Cu (or CuPT), and the fungicides dichlofluanid and tolylfluanid were irgarol and Cu mediated P450 induction leading to faster activation of dichlofluanid and tolylfluanid. But this hypothesis would have to be tested.diuron and TCMTB also induced synergy together with irgarol in two cases each, in the heterotrophic organisms, but information in terms of possible P450 induced activation of these two compounds has not been found.

Metal induced P450 activity could possibly also play a role in the synergies with the phosphorothioate organophosphates malathion, chlorpyriphos and dimethoate [56], [82](Sejerøe 2011) (Table S4 in File S1), but as synergies between metal ions an phosphate organophosphates as dichlorvos, the carbamate carbofuran and the azole penconazole [56]Sejerøe 2011) was also found, other mechanisms are most likely also of importance. Lister et al (2011) found an increased uptake and metabolization rate of chlorpyriphos in the presence of Ni, but data were two variable to say anything definite [83]. However, as both P450 monooxygenases and esterases are important enzymes in many biochemical processes, changes in their activity could also affect uptake, excretion or possibly some of the mechanisms used for inactivation of metals in different organisms, though these hypotheses must be subjected to experimental scrutiny.

### Is Synergy of any Importance in Nature?

For synergistic interactions to take place in the environment, interacting chemicals have both to co-occur and to be present at levels high enough to induce the synergy. Co-occurrence does happen, as has been shown for both pesticides and antifoulants [11], [59], [84]. Looking at the cases presented in this review, however, most experiments showing significant synergy use chemical concentrations in the high µg L^−1^ to mg L^−1^ range, which is considerably above the concentrations most often monitored in the environment (pg L^−1^ to the low µg L^−1^ range) [11], [59], [84]. Very few studies though use realistic concentration ranges, Laetz et al (2009) being such an exception. It is, however, likely that a threshold for synergistic interactions exists for most synergists, and that only a few proven synergists will act as synergists at any endpoint when diluted down to realistic environmental levels. This loss of efficiency as a synergist has for example been shown for piperonyl butoxide (PBO), a known P450 inhibitor, when used to formulate pyrethroid insecticides for mosquito control [85]. In this case, adding the synergist to the aquatic environment did not increase the efficacy of the insecticide towards an aquatic crustacean. Another case, however, showed PBO to enhance pyrethroid toxicity down to concentrations as low as 25 µg L^−1^ [86]. Hence, more data is needed to determine if a lower threshold for synergists interfering with metabolic processes do exist. In these studies it will be important to include sub-lethal endpoints such as growth and reproduction so that true long term effects on population growth can be estimated.

## Conclusion

From the present review of possible mechanisms causing the observed synergies, it can be concluded that interactions on metabolic processes affecting the transformation of xenobiotics seem to be far the most common mechanism of synergy, though interactions on availability and uptake might play an important role for metal/metal synergies. For the synergistic interactions between pesticides, with cholinesterase inhibitors and azole fungicides being present in 95% of the described synergistic cases, the chemical groups causing synergy can be well defined. For the antifoulants the pattern was less clear, primarily due to the lack of knowledge on the interference of the compounds with metabolic processes. However, knowing that most synergistic interactions most likely stem from interactions on metabolic processes, it would be possible to screen for potential synergists using either *in vitro* assays on P450 monooxygenase or esterase inhibition potential, or by investigating metabolization kinetics *in vivo* in representative test species; though the latter is quite labour intensive.

In the introduction it was stated that if we could identify the groups of chemicals that are likely to induce synergistic interactions, special precautions could be taken in the risk assessment of these chemicals. The present review shows that some groups of potential synergists can indeed be identified, while others need more research to be specifically defined as synergists.

That said, considering the generally high chemical concentrations needed to induce synergistic interactions, their importance as synergists within naturally occurring exposure scenarios is most likely of a relatively small importance compared to the additive effect of many co-occuring pollutants. Even if one compound enhances the effect of another compound four-fold, it only takes another three compounds of a similar strength to arrive at the same joint toxicity. And considering the complex pollution patterns monitored [13], [33], [87], the additive effect of the many co-occurring pollutants might likely project a larger hazard than those of the presence of a few synergist. Hence, in a regulatory perspective addressing the cumulative effect of co-occurring chemicals is the first and most important step in providing a more realistic hazard assessment of chemical cocktails in both man and environment.

## Supporting Information

Checklist S1 **PRISMA 2009 checklist for systematic reviews and meta-analyses.**
(DOC)

File S1 Table S1A. Antagonistic and additive pesticide mixtures. All binary antagonistic and concentration additive pesticide mixtures from Belden et al (2007) sorted with increasing Model Deviation ration (MDR). The synergistic mixtures from Belden et al (2007) are included in Table S1B. For information on species tested, endpoint and original references, please see Belden et al (2007), Supplementary material, Table 1. Table S1B. Synergistic pesticide mixtures. The mixtures are sorted with increasing MDR and including information on the test species, its phylum, sub-phylum or class, the endpoint tested and the reference of the original study. The synergistic mixtures also included in Belden et al (2007) are given in bold. In the cases where the same mixtures were repeated on the same organism in independent experiments, MDR-values are given for all experiments and are sorted according to the highest MDR value. One full ray-design is defined as one experiment, even though several mixture ratios were tested. Table S2. Metal mitures. Antagonistic and concentration additive mixtures of metal ions from Vijvers et al (2011) and Xu et al (2011) from which MDR-values could be calculated, sorted with the binary mixtures first and then with increasing MDR. Below are the four synergistic mixtures found of which one mixture, given in bold, was obtained from Vijvers et al (2011). The table includes information on the test species, its phylum, sub-phylum or class, the endpoint tested and the reference of the original study. The last three entries are the three extra synergistic mixtures found by the additional database study. Table S3. Mixtures of antifoulants. All mixtures of antifoulants (Antif) from which MDR-values could be calculated, sorted with the binary mixtures first and then with increasing MDR. The table includes information on the test species, its phylum, sub-phylum or class, the endpoint tested and the reference of the original study. For full chemical names and chemical class and mode of action of the antifaulants, please consult Table 1 in the manuscript. The following names are abbreviated: Irgarol1051 (Irgarol), Seanine211 (Seanine), Chlorothalonil (Chlorot.), Dichlofluanid (Dichlo), Tolylfuanid (Tolyl). Table S4. Additional synergistic mixtures. Synergistic mixtures between metals and organic compounds which did not fit into any of the three categories; pesticides, metals or antifoulants, sorted with increasing MDR. The table includes information on the test species, its phylum, sub-phylum or class, the endpoint tested and the reference of the original study.
(DOCX)
