# Supplementary material for: Quantifying Synergy: A Systematic Review of Mixture Toxicity Studies within Environmental Toxicology
Source: PLoS One. 2014 May 2;9(5):e96580. doi: 10.1371/journal.pone.0096580 (PMC4008607; doi:10.1371/journal.pone.0096580)
Supplement: File S1 — Table S1A. Antagonistic and additive pesticide mixtures. All binary antagonistic and concentration additive pesticide mixtures from Belden et al (2007) sorted with increasing Model Deviation ration (MDR). The synergistic mixtures from Belden et al (2007) are included in Table S1B. For information on species tested, endpoint and original references, please see Belden et al (2007), Supplementary material, Table 1. Table S1B. Synergistic pesticide mixtures. The mixtures are sorted with increasing MDR and including information on the test species, its phylum, sub-phylum or class, the endpoint tested and the reference of the original study. The synergistic mixtures also included in Belden et al (2007) are given in bold. In the cases where the same mixtures were repeated on the same organism in independent experiments, MDR-values are given for all experiments and are sorted according to the highest MDR value. One full ray-design is defined as one experiment, even though several mixture ratios were tested. Table S2. Metal mitures. Antagonistic and concentration additive mixtures of metal ions from Vijvers et al (2011) and Xu et al (2011) from which MDR-values could be calculated, sorted with the binary mixtures first and then with increasing MDR. Below are the four synergistic mixtures found of which one mixture, given in bold, was obtained from Vijvers et al (2011). The table includes information on the test species, its phylum, sub-phylum or class, the endpoint tested and the reference of the original study. The last three entries are the three extra synergistic mixtures found by the additional database study. Table S3. Mixtures of antifoulants. All mixtures of antifoulants (Antif) from which MDR-values could be calculated, sorted with the binary mixtures first and then with increasing MDR. The table includes information on the test species, its phylum, sub-phylum or class, the endpoint tested and the reference of the original study. For full chemical names and chemical [file pone.0096580.s002.docx]

**Table S1A. Antagonistic and additive pesticide mixtures.** All binary antagonistic and concentration additive pesticide mixtures from Belden et al (2007) [1] sorted with increasing Model Deviation ration (MDR). The synergistic mixtures from Belden et al (2007) are included in Table S1B. For information on species tested, endpoint and original references, please see Belden et al (2007), Supplementary material, Table 1.

| **Pesticide 1** | **Pesticide 2** | **MDR** | **Test organism** | **Phylum/Subphylum or Class** | **Endpoint** | **Reference** |
| --- | --- | --- | --- | --- | --- | --- |
| Amitrol | Glufosinate | 0.18 | - | Chlorophyta | - | - |
| Mesotrione | Acifluorfen | 0.3 | - | Liliopsida | - | - |
| Prochloraz | 2.4-D | 0.36 | - | Chlorophyta | - | - |
| Mesotrione | Metsulfuron-methyl | 0.42 | - | Liliopsida | - | - |
| Mesotrione | Mecoprop | 0.42 | - | Liliopsida | - | - |
| Mesotrione | Metsulfuron-methyl | 0.44 | - | Liliopsida | - | - |
| Atrazine | Mevinphos | 0.44 | - | Arthropodae/Insecta | - | - |
| 2.4-D monosodium | Methanearsonate | 0.46 | - | Arthropodae/Crustacea | - | - |
| Anilazine | Prochloraz | 0.48 | - | Chlorophyta | - | - |
| Mecoprop | Glyphosate | 0.49 | - | Liliopsida | - | - |
| Prochloraz | Dimethoate | 0.49 | - | Arthropodae/Crustacea | - | - |
| Bentazone | Metazachlor | 0.5 | - | Chlorophyta | - | - |
| Mesotrione | Acifluorfen | 0.5 | - | Liliopsida | - | - |
| Hexacarbamate | Baythion | 0.51 | - | Cordata/Osteichthyes | - | - |
| Parathion | 2.4-D | 0.53 | - | Chlorophyta | - | - |
| Acephate | Fenvalerate | 0.54 | - | Cordata/Osteichthyes | - | - |
| Lindane | Bentazone | 0.54 | - | Chlorophyta | - | - |
| Profenophos | Endosulfan | 0.55 | - | Arthropodae/Crustacea | - | - |
| Anilazine | Parathion | 0.55 | - | Chlorophyta | - | - |
| Prochloraz | Terbuthylazine | 0.56 | - | Liliopsida | - | - |
| Metsulfuron-methyl | Terbuthylazine | 0.57 | - | Liliopsida | - | - |
| Mecoprop | Mesotrion | 0.57 | - | Liliopsida | - | - |
| Mesothroin | Mecoprop | 0.58 | - | Liliopsida | - | - |
| Glyphosate | Terbuthylazine | 0.58 | - | Liliopsida | - | - |
| Anilazine | Bentazone | 0.58 | - | Chlorophyta | - | - |
| Anilazine | 2.4-D | 0.59 | - | Chlorophyta | - | - |
| Lindane | Metazachlor | 0.59 | - | Chlorophyta | - | - |
| Prochloraz | Diquat | 0.59 | - | Liliopsida | - | - |
| Mesotrione | Metsulfuron-methyl | 0.6 | - | Chlorophyta | - | - |
| Mesotrione | Acifluorfen | 0.6 | - | Liliopsida | - | - |
| Trichlorfon | Ru-11679 | 0.6 | - | Cordata/Osteichthyes | - | - |
| Prochloraz | Acifluorfen | 0.6 | - | Chlorophyta | - | - |
| Atrazine | Methylazinphos | 0.6 | - | Arthropodae/Insecta | - | - |
| Acifluorfen | Diquat | 0.61 | - | Liliopsida | - | - |
| Glyphosate | Terbuthylazine | 0.62 | - | Chlorophyta | - | - |
| Metsulfuron-methyl | Terbuthylazine | 0.62 | - | Liliopsida | - | - |
| Acifluorfen | Diquat | 0.62 | - | Liliopsida | - | - |
| Glyphosate | Terbuthylazine | 0.62 | - | Liliopsida | - | - |
| Mecoprop | Terbuthylazine | 0.62 | - | Liliopsida | - | - |
| Mecoprop | Mesotrion | 0.62 | - | Liliopsida | - | - |
| Mecoprop | Terbuthylazine | 0.63 | - | Liliopsida | - | - |
| Copper | Methylazinphos | 0.64 | - | Arthropodae/Crustacea | - | - |
| Acifluorfen | Diquat | 0.65 | - | Chlorophyta | - | - |
| Methylparathion | Copper | 0.65 | - | Arthropodae/Crustacea | - | - |
| Trichlorfon | Pyrethrum | 0.66 | - | Cordata/Osteichthyes | - | - |
| Baythion | Resmethrin | 0.66 | - | Cordata/Osteichthyes | - | - |
| Trichlorfon | Resmethrin | 0.68 | - | Cordata/Osteichthyes | - | - |
| Prochloraz | Acifluorfen | 0.68 | - | Liliopsida | - | - |
| Mexacarbate | Pyrethrum | 0.69 | - | Cordata/Osteichthyes | - | - |
| Trichlorfon | Mexacarbate | 0.69 | - | Cordata/Osteichthyes | - | - |
| Mexacarbate | Azinophos-methyl | 0.69 | - | Cordata/Osteichthyes | - | - |
| Metsulfuron-methyl | Terbuthylazine | 0.7 | - | Chlorophyta | - | - |
| Mesotrione | Glyphosate | 0.7 | - | Liliopsida | - | - |
| Mesotrione | Metsulfuron-methyl | 0.72 | - | Liliopsida | - | - |
| Pyrethrum | Azinophos-methyl | 0.72 | - | Cordata/Osteichthyes | - | - |
| Prochloraz | Azoxystrobin | 0.72 | - | Chlorophyta | - | - |
| Metsulfuron-methyl | Terbuthylazine | 0.73 | - | Liliopsida | - | - |
| 2.4-D | Trichlopyr | 0.74 | - | Cordata/Osteichthyes | - | - |
| Mecoprop | Glyphosate | 0.76 | - | Liliopsida | - | - |
| Mecoprop | Terbuthylazine | 0.76 | - | Liliopsida | - | - |
| Acephate | Fenvalerate | 0.78 | - | Cordata/Osteichthyes | - | - |
| Diazinon | Methidathion | 0.79 | - | Arthropodae/Insecta | - | - |
| Glyphosate | mecoprop | 0.79 | - | Liliopsida | - | - |
| Prochloraz | Simazine | 0.79 | - | Chlorophyta | - | - |
| Chlortoluron | Metazachlor | 0.8 | - | Chlorophyta | - | - |
| Resmethrin | Azinophos-methyl | 0.8 | - | Cordata/Osteichthyes | - | - |
| 2.4-D | Glyphosate | 0.81 | - | Cordata/Osteichthyes | - | - |
| Mexacarbate | Resmethrin | 0.81 | - | Cordata/Osteichthyes | - | - |
| Metsulfuron-methyl | triasulfuron | 0.82 | - | Liliopsida | - | - |
| 2.4-D Buthyl Ester | Picloram | 0.84 | - | Cordata/Osteichthyes | - | - |
| Parathion | Glyphosate | 0.84 | - | Chlorophyta | - | - |
| Azinophos-methyl | chlorpyriphos | 0.85 | - | Arthropodae/Insecta | - | - |
| 2,4-D | methabenzthiazuron | 0.86 | - | Chlorophyta | - | - |
| Metsulfuron-methyl | Terbuthylazine | 0.86 | - | Liliopsida | - | - |
| Mesotrione | Metsulfuron-methyl | 0.86 | - | Liliopsida | - | - |
| Diazinon | Chlorpyriphos | 0.88 | - | Arthropodae/Crustacea | - | - |
| Glyphosate | Simazine | 0.88 | - | Chlorophyta | - | - |
| Glyphosate | Mecoprop | 0.88 | - | Liliopsida | - | - |
| Ru-11679 | Azinophos-methyl | 0.88 | - | Cordata/Osteichthyes | - | - |
| Glyphosate | Tri-allate | 0.89 | - | Chlorophyta | - | - |
| Pyrethrum | Baythion | 0.89 | - | Cordata/Osteichthyes | - | - |
| Prochloraz | Tri-allate | 0.89 | - | Chlorophyta | - | - |
| Bentazone | Chlortoluron | 0.9 | - | Chlorophyta | - | - |
| prochloraz | Lindane | 0.9 | - | Chlorophyta | - | - |
| Glyphosate | Metsulfuron-methyl | 0.91 | - | Chlorophyta | - | - |
| Simazine | Tri-allate | 0.91 | - | Chlorophyta | - | - |
| Chlortoluron | 2.4-D | 0.92 | - | Chlorophyta | - | - |
| Ru-11679 | Mexacarbate | 0.92 | - | Cordata/Osteichthyes | - | - |
| Prochloraz | Diquat | 0.92 | - | Chlorophyta | - | - |
| Lindane | Tri-allate | 0.92 | - | Chlorophyta | - | - |
| Azinophos-methyl | Methidathion | 0.93 | - | Arthropodae/Insecta | - | - |
| Mecoprop | Glyphosate | 0.93 | - | Liliopsida | - | - |
| Lindane | Glyphosate | 0.93 | - | Chlorophyta | - | - |
| Thrichlorfon | Baythion | 0.94 | - | Cordata/Osteichthyes | - | - |
| Bentazone | Glyphosate | 0.94 | - | Chlorophyta | - | - |
| 2,4-D | Simazine | 0.94 | - | Chlorophyta | - | - |
| Azinophos-methyl | Fenvalerate | 0.94 | - | Cordata/Osteichthyes | - | - |
| Anilazine | Simazine | 0.94 | - | Chlorophyta | - | - |
| Prochloraz | Parathion | 0.94 | - | Chlorophyta | - | - |
| Bentazone | methabenzthiazuron | 0.96 | - | Chlorophyta | - | - |
| Anilazine | Chlortolurone | 0.96 | - | Chlorophyta | - | - |
| Atrazine | Alachlor | 0.97 | - | Cordata/Osteichthyes | - | - |
| Terbuthylazine | Diquat | 0.98 | - | Chlorophyta | - | - |
| Carbofuran | Methylparathion | 1 | - | Arthropodae/Crustacea | - | - |
| Methylparathion | Methoxychlor | 1 | - | Arthropodae/Insecta | - | - |
| Glyphosate | Trichlopyr | 1 | - | Cordata/Osteichthyes | - | - |
| Lindane | Methanbenzthiazuron | 1 | - | Chlorophyta | - | - |
| Anilazine | Methanbenzthiazuron | 1.01 | - | Chlorophyta | - | - |
| Diazinon | chlorpyriphos | 1.03 | - | Arthropodae/Insecta | - | - |
| Chlortoluron | methabenzthiazuron | 1.03 | - | Chlorophyta | - | - |
| Atrazine | Chlorpyriphos | 1.04 | - | Chlorophyta | - | - |
| Atrazine | Simazine | 1.05 | - | Chlorophyta | - | - |
| Mesotrione | Glyphosate | 1.05 | - | Liliopsida | - | - |
| Prochloraz | Methanbenzthiazuron | 1.05 | - | Chlorophyta | - | - |
| Lindane | 2.4-D | 1.06 | - | Chlorophyta | - | - |
| Bentazone | 2,4-D | 1.07 | - | Chlorophyta | - | - |
| chlorpyriphos | Methidathion | 1.09 | - | Arthropodae/Insecta | - | - |
| Ru-11679 | pyrethrum | 1.09 | - | Cordata/Osteichthyes | - | - |
| Prochloraz | Glyphosate | 1.09 | - | Chlorophyta | - | - |
| Anilazine | Lindane | 1.09 | - | Chlorophyta | - | - |
| Atrazine | Metribuzin | 1.1 | - | Chlorophyta | - | - |
| Chlorpyriphos | Esfenvalerate | 1.1 | - | Arthropodae/Insecta | - | - |
| Prochloraz | Bentazone | 1.1 | - | Chlorophyta | - | - |
| Terbuthylazine | Diquat | 1.11 | - | Liliopsida | - | - |
| Bentazone | Simazine | 1.12 | - | Chlorophyta | - | - |
| Chlortoluron | Glyphosate | 1.12 | - | Chlorophyta | - | - |
| Mesotrione | Mecoprop | 1.12 | - | Liliopsida | - | - |
| Lindane | Simazine | 1.12 | - | Chlorophyta | - | - |
| Metsulfuron-methyl | triasulfuron | 1.14 | - | Chlorophyta | - | - |
| Chlortoluron | Tri-allate | 1.14 | - | Chlorophyta | - | - |
| Glyphosate | Metazachlor | 1.14 | - | Chlorophyta | - | - |
| Prochloraz | Chlortoluron | 1.14 | - | Chlorophyta | - | - |
| MCPA | mecoprop | 1.15 | - | Liliopsida | - | - |
| pyretrum | resmethrin | 1.15 | - | Cordata/Osteichthyes | - | - |
| 2,4-D | Metazachlor | 1.15 | - | Chlorophyta | - | - |
| Thiobencarb | Molinate | 1.16 | - | Arthropodae/Crustacea | - | - |
| Diazinon | Azinophos-methyl | 1.16 | - | Arthropodae/Insecta | - | - |
| 2,4-D | Tri-allate | 1.16 | - | Chlorophyta | - | - |
| Methylparathion | Methylazinphos | 1.2 | - | Arthropodae/Crustacea | - | - |
| Lindane | Chlortoluron | 1.2 | - | Chlorophyta | - | - |
| Parathion | Bentazone | 1.2 | - | Chlorophyta | - | - |
| Parathion | Chlortoluron | 1.2 | - | Chlorophyta | - | - |
| Copper | Dichlorvos | 1.2 | - | Arthropodae/Crustacea | - | - |
| Anilazine | Metazachlor | 1.22 | - | Chlorophyta | - | - |
| Bentazone | Tri-allate | 1.24 | - | Chlorophyta | - | - |
| Chlortoluron | Simazine | 1.27 | - | Chlorophyta | - | - |
| Ru-11679 | resmethrin | 1.28 | - | Cordata/Osteichthyes | - | - |
| Parathion | Simazine | 1.28 | - | Chlorophyta | - | - |
| Atrazine | Alachlor | 1.31 | - | Cordata/Osteichthyes | - | - |
| Parathion | Metazachlor | 1.31 | - | Chlorophyta | - | - |
| Metazachlor | methabenzthiazuron | 1.33 | - | Chlorophyta | - | - |
| Atrazine | Alachlor | 1.34 | - | Amphibia | - | - |
| Azinophos-methyl | Endosulfan | 1.35 | - | Cordata/Osteichthyes | - | - |
| Prochloraz | Terbuthylazine | 1.37 | - | Chlorophyta | - | - |
| Glyphosate | methabenzthiazuron | 1.4 | - | Chlorophyta | - | - |
| Diazinon | Esfenvalerate | 1.4 | - | Cordata/Osteichthyes | - | - |
| Thrichlorfon | Azinophos-methyl | 1.42 | - | Cordata/Osteichthyes | - | - |
| Metazachlor | Simazine | 1.42 | - | Chlorophyta | - | - |
| Atrazine | Alachlor | 1.43 | - | Amphibia | - | - |
| methabenzthiazuron | Simazine | 1.43 | - | Chlorophyta | - | - |
| methabenzthiazuron | Tri-allate | 1.46 | - | Chlorophyta | - | - |
| Azinophos-methyl | Fenvalerate | 1.47 | - | Cordata/Osteichthyes | - | - |
| Chlorpyriphos | Esfenvalerate | 1.5 | - | Cordata/Osteichthyes | - | - |
| Prochloraz | Metazachlor | 1.5 | - | Chlorophyta | - | - |
| Mesotrione | Glyphosate | 1.51 | - | Liliopsida | - | - |
| Metazachlor | Tri-allate | 1.56 | - | Chlorophyta | - | - |
| Parathion | Methanbenzthiazuron | 1.61 | - | Chlorophyta | - | - |
| Anilazine | Glyphosate | 1.64 | - | Chlorophyta | - | - |
| Ru-11679 | Baythion | 1.67 | - | Cordata/Osteichthyes | - | - |
| Atrazine | Alachlor | 1.68 | - | Amphibia | - | - |
| Parathion | Tri-allate | 1.68 | - | Chlorophyta | - | - |
| Atrazine | Methyl-parathion | 1.69 | - | Arthropodae/Insecta | - | - |
| Atrazine | Methyl-parathion | 1.69 | - | Arthropodae/Insecta | - | - |
| Carbofuran | Malathion | 1.7 | - | Arthropodae/Crustacea | - | - |
| Atrazine | Chlorpyriphos | 1.72 | - | Arthropodae/Insecta | - | - |
| Azinophos-methyl | Endosulfan | 1.75 | - | Cordata/Osteichthyes | - | - |
| Lindane | Parathion | 1.79 | - | Chlorophyta | - | - |
| Copper | Carbofuran | 1.8 | - | Arthropodae/Crustacea | - | - |
| Atrazine | Chlorot. | 1.81 | - | Chlorophyta | - | - |
| Prochloraz | Diquat | 1.85 | - | Arthropodae/Crustacea | - | - |
| Atrazine | Alachlor | 1.89 | - | Amphibia | - | - |

**Table S1B. Synergistic pesticide mixtures**. The mixtures are sorted with increasing MDR and including information on the test species, its phylum, sub-phylum or class, the endpoint tested and the reference of the original study. The synergistic mixtures also included in Belden et al (2007) are given in bold. In the cases where the same mixtures were repeated on the same organism in independent experiments, MDR-values are given for all experiments and are sorted according to the highest MDR value. One full ray-design [2] is defined as one experiment, even though several mixture ratios were tested.

| **Pesticide 1** | **Pesticide 2** | **MDR** | **Test organism** | **Phylum/Subphylum or Class** | **Endpoint** | **Reference** |
| --- | --- | --- | --- | --- | --- | --- |
| Dimethoate | chlorpyriphos | ~2* | *Chironomous dilutus* | Arthropodae/Insecta | Mobility (96h) | LeBlanc et al, 2012 [3] |
| Dimethoate | Lindane | ~2* | *Folsomia candida* | Arthropodae/Insecta | Mortality (28d) | Amorim et al 2012 [4] |
| Azinphos-methyl | chlorpyriphos | ~2* | *Planorbarius corneus* | Gastropodae |  | Cacciatore et al. 2012 [5] |
| Hexazinon | Methidathion | 2.0 | *Chironomus tentans* | Arthropodae/Insecta | Mobility (96h) | Lydy & Austin 2004 [6] |
| Acrinathrin | Carbofuran | 2.0 | *Frankliniella occidentalis* | Arthropodae/Insecta | Mortality (24h) | Bielza *et al.* 2007 [7] |
| Chlorpyriphos | imidachloprid | >2* | *Chironomous dilutus* | Arthropodae/Insecta | Mobility (96h) | LeBlanc et al, 2012 [3] |
| Diazinon | chlorpyriphos | 2.2 | *Oncorhynchus sp.* | Cordata/Osteichthyes | AChE activity | Laetz et al 2009 [8] |
| **Diquat** | **Endothall** | **2.2** | ***Carassius auratus*** | **Cordata/Osteichthyes** | **Mortality (96h)** | **Berry 1984 [9]** |
| Cyanazin | Chlorpyrifos | 2.2 | *Chironomus tentans* | Arthropodae/Insecta | Mortality (48h) | Jin-Clark *et al.* 2002 [10] |
| Cyanazin | Diazinon | 2.2 | *Chironomus tentans* | Arthropodae/Insecta | Mobility (96h) | Lydy & Austin 2004 [6] |
| Cyanozin | Diazinon | 2.2 | *Chironomus tentans* | Arthropodae/Insecta | Mobility (96h) | Schuler *et al.* 2005 [11] |
| Alfacypermethrin | Fenpropidin | 2.2 | *Daphnia magna* | Arthropodae/Crustacea | Mobility (48h) | Nørgaard and Cedergreen, 2010[12] |
| Permethrin | Propoxur | 2.2 | *Culex quinquefasciatus* | Arthropodae/Insecta | Mortality (24h) | Corbel *et al.* 2003 [13] |
| Azoxystrobin | thiram | 2.3 | *Sclerotinia sclerotiorum* | Ascomycota | Mycelia Growth | Duan et al 2012 [14] |
| Simazin | Methidathion | 2.4 | *Chironomus tentans* | Arthropodae/Insecta | Mobility (96h) | Lydy & Austin 2004 [6] |
| Chlorpyriphos | Imidacloprid | 2.3 | *Caenorhabditis elegans* | Nematodae/Chromadorae | Reproduction (24h) | Svendsen et al 2010 [15] |
| Atrazin | Diazinon | 2.4 | *Chironomus tentans* | Arthropodae/Insecta | Mobility (96h) | Schuler *et al.* 2005 [11] |
| Prochloraz | Acifluorfen | 2.4 | *Vibrio Fisheri* | Protobacteria | Luminescence (30 min) | Cedergreen *et al.* 2008 [16] |
| Simazin | Diazinon | 2.5 | *Chironomus tentans* | Arthropodae/Insecta | Mobility (96h) | Schuler *et al.* 2005 [11] |
| Chlorpyriphos | Esfenvalerate | 2.5 | *Pimephales promelas* | Cordata/Osteichthyes | Mobility (96h) | Belden & Lydy 2006 [17] |
| Acrinathrin | Formetanat | 2.5 | *Frankliniella occidentalis* | Arthropodae/Insecta | Mortality (24h) | Bielza *et al.* 2007 [7] |
| tau-fluvalinate | Coumaphos | 2.1; 2.7 | *Apis mellifera* | Arthropodae/Insecta | Mortality (24h) | Johnson et al, 2009 [18] |
| Monocrotophos | Pirimicarb | 2.7 | *Tilapia nilotica* | Cordata/Osteichthyes | Mortality (96h) | Abdel-Nasser 1991 [19] |
| **Atrazin** | **Diazinon** | **2.7** | ***Chironomus tentans*** | **Arthropodae/Insecta** | **Mobility (96h)** | **Belden & Lydy 2000 [20]** |
| Chlorpyriphos | Penconazole | 2.73; 2.76 | *Ceaenorhabditis elegans* | Nematoda/Chromadorae | Reproduction (96h) | Sejerøe 2011 [21] |
| **Atrazin** | **Malathion** | **2.8** | ***Chironomus tentans*** | **Arthropodae/Insecta** | **Mobility (96h)** | **PapeLindstrom & Lydy 1997 [22]** |
| **Methidathion** | **Phosphamidon** | **2.8** | ***Homarus americanus*** | **Arthropodae/Crustacea** | **Mortality (48h)** | **McLeese & Metcalfe 1979 [23]** |
| **Atrazin** | **Chlorpyrifos** | **2.8** | ***Hyallella azteca*** | **Arthropodae/Crustacea** | **Mobility (96h)** | **Anderson & Lydy 2002 [24]** |
| Prochloraz | Azoxystrobin | 2.9; 2.9 | *Vibrio Fisheri* | Protobacteria | Luminescence (30 min) | Cedergreen *et al.* 2008 [16] |
| Carbofuran | Malathion | 2.9 | *Oncorhynchus sp.* | Cordata/Osteichthyes | AChE activity | Laetz et al 2009 [8] |
| **Atrazin** | **Methylparathion** | **2.9** | ***Hyallella azteca*** | **Arthropodae/Crustacea** | **Mobility (96h)** | **Anderson & Lydy 2002 [24]** |
| Formetanat | methiocarb | 2.9 | *Frankliniella occidentalis* | Arthropodae/Insecta | Mortality (24h) | Bielza *et al.* 2007 [7] |
| Atrazin | Diazinon | 3.0 | *Hyallella azteca* | Arthropodae/Crustacea | Mobility (96h) | Anderson & Lydy 2002 [24] |
| **Phenthoat** | **Carbaryl** | **3.2** | ***Channa punctata*** | **Cordata/Osteichthyes** | **Mortality (48h)** | **Rao *et al.* 1985 [25]** |
| Chlorpyriphos | Thiacloprid | 3.3 | *Caenorhabditis elegans* | Nematoda/Chromadorae | Reproduction (24h) | Svendsen et al. 2010 [15] |
| Prochloraz | Diquat | 3.5 | *Daphnia magna* | Arthropodae/Crustacea | Mortality (48h) | Cedergreen *et al.* 2008 [16] |
| **Anilazin** | **Tri-allat** | **3.5** | ***Scenedesmus vacuolatus*** | **Chlorophyta** | **Reproduction (24h)** | **Faust *et al.* 1994 [26]** |
| **Chlorpyriphos** | **Endosulfan** | **3.6** | ***Ceriodaphnia dubia*** | **Arthropodae/Crustacea** | **Mortality (48h)** | **Woods *et al.* 2002 [27]** |
| Prochloraz | Azoxystrobin | 2.2; 2.4; 3.6 | *Daphnia magna* | Arthropodae/Crustacea | Mortality (48h) | Cedergreen *et al.* 2008 [16] |
| Carbaryl | Malathion | 3.8 | *Oncorhynchus sp.* | Cordata/Osteichthyes | AChE activity | Laetz et al 2009 [8] |
| **Atrazin** | **Trichlorfon** | **3.9** | ***Chironomus tentans*** | **Arthropodae/Insecta** | **Mobility (96h)** | **PapeLindstrom & Lydy 1997 [22]** |
| **Chlorpyriphos** | **Profenofos** | **4.0** | ***Ceriodaphnia dubia*** | **Arthropodae/Crustacea** | **Mortality (48h)** | **Woods *et al.* 2002 [27]** |
| Atrazin | Chlorpyrifos | 4.0 | *Chironomus tentans* | Arthropodae/Insecta | Mobility (96h) | Belden & Lydy 2000 [20] |
| Atrazin | Chlorpyrifos | 4.0 | *Eisenia fetida* | Annelida/Clitelata | Mortality (96h) | Lydy & Linck 2003 [28] |
| Acrinathrin | Carbosulfan | 4.0 | *Frankliniella occidentalis* | Arthropodae/Insecta | Mortality (24h) | Bielza *et al.* 2007 [7] |
| Fenvalerat | Acephat | 4.2 | *Fundulus heteroclitus* | Cordata/Osteichthyes | Mortality (96h) | Fulton & Scott 1991 [29] |
| Lambdacyhalothrin | Penconazol | 4.4 | *Apis mellifera* | Arthropodae/Insecta | Mortality (24h) | Pilling & Jepson 1993 [30] |
| Diazinon | Thiacloprid | 4.4 | *Caenorhabditis elegans* | Nematoda/Chromadorae | Reproduction (24h) | Svendsen et al. 2010 [15] |
| Diuron | Methidathion | 4.8 | *Chironomus tentans* | Arthropodae/Insecta | Mobility (96h) | Lydy & Austin 2004 [6] |
| Esfenvalerat | Prochloraz | 4.0; 5.0 | *Daphnia magna* | Arthropodae/Crustacea | Mortality (48h) | Cedergreen *et al.* 2008 [16] |
| Acrinathrin | methiocarb | 4.3; 5.1 | *Frankliniella occidentalis* | Arthropodae/Insecta | Mortality (24h) | Bielza *et al.* 2007 [7] |
| Prochloraz | Esfenvalerate | 6.0 | *Daphnia magna* | Arthropodae/Crustacea | Mortality (48h) | Cedergreen *et al.* 2008 [16] |
| Lambdacyhalothrin | Imazalil | 7.2 | *Apis mellifera* | Arthropodae/Insecta | Mortality (24h) | Pilling & Jepson 1993 [30] |
| Lambdacyhalothrin | Triadimenol | 7.6 | *Apis mellifera* | Arthropodae/Insecta | Mortality (24h) | Pilling & Jepson 1993 [30] |
| Alfacypermethrin | Epoxiconazol | 5.5; 7.6 | *Daphnia magna* | Arthropodae/Crustacea | Mobility (48h) | Nørgaard & Cedergreen 2010 [12] |
| Malathion | Dioxathion | 8.2 | *Salmo gairdneri* | Cordata/Osteichthyes | - | Marking & Dawson 1975 [31] |
| Carbofuran | Malathion | 8.2 | *Oncorhynchus sp.* | Cordata/Osteichthyes | AChE activity | Laetz et al 2009 [8] |
| Lambdacyhalothrin | Prochloraz | 9.1 | *Apis mellifera* | Arthropodae/Insecta | Mortality (24h) | Pilling & Jepson 1993 [30] |
| Alfacypermethrin | Propiconazol | 5.6; 9.1 | *Daphnia magna* | Arthropodae/Crustacea | Mobility (48h) | Nørgaard & Cedergreen 2010 [12] |
| **Phenthoat** | **Quinalphos** | **9.6** | ***Oreochromis mossambicus*** | **Cordata/Osteichthyes** | **Mortality (96h)** | **Durairaj & Selvarajan 1995 [32]** |
| Malathion | chlorpyriphos | 10.4 | *Oncorhynchus sp.* | Cordata/Osteichthyes | AChE activity | Laetz et al 2009 [8] |
| Diazinon | Malathion | 10.4 | *Oncorhynchus sp.* | Cordata/Osteichthyes | AChE activity | Laetz et al 2009 [8] |
| Lambdacyhalothrin | Triadimefon | 11.5 | *Apis mellifera* | Arthropodae/Insecta | Mortality (24h) | Pilling & Jepson 1993 [30] |
| Alfacypermethrin | Prochloraz | 12.5; 16.1 | *Daphnia magna* | Arthropodae/Crustacea | Mobility (48h) | Nørgaard & Cedergreen 2010 [12] |
| Lambdacyhalothrin | Propiconazol | 16.2 | *Apis mellifera* | Arthropodae/Insecta | Mortality (24h) | Pilling & Jepson 1993 [30] |
| Prochloraz | Diquat | 2.0; 16.7 | *Vibrio fischeri* | Protobacteria | Luminescence (30 min) | Cedergreen *et al.* 2008 [16] |
| Diazinon | Imidacloprid | 17.4 | *Caenorhabditis elegans* | Nematoda/Chromadorae | Reproduction (24h) | Svendsen et al. 2010 [15] |
| Deltamethrin | Carbaryl | 20.0 | *Lymnaea acuminata* | Mollusca/gastropoda | Mortality (96h) | Sahay & Agarwal 1997 [33] |
| **Dichlorvos** | **Malathion** | **143.0** | ***Tigriopus brevicornis*** | **Arthropodae/Crustacea** | **Mortality (96h)** | **Forget *et al.* 1999 [34]** |
|  |  |  |  |  |  |  |
| **Mixtures including more than two pesticides** | | |  |  |  |  |
| Chlorpyriphos | diazonon, dimethoate, aceptate and malathion | 1.2-2.1 | Male rats | \| Cordata/Mammalia \| \| --- \| | ChE activity, motor activity and gait score | Moser et al 2005 [35] |
| Chlorpyriphos | , diazonon, dimethoate, aceptate and malathion, | 1.3-3.5 | Rats | Cordata/Mammalia | Rotarod performance | Moser et al 2006 [36] |
| **Chlorpyriphos** | **endosulfan, profenofos** | **4.2** | ***Ceriodaphnia dubia*** | **Arthropodae/Crustacea** | **Mortality (48h)** | **Woods *et al.* 2002 [27]** |
| **Acetochlor** | **Alachlor, butachlor, dimethachlor, metazachlor, metolachlor, propachlor, pretilachlor** | **4.4** | ***Scenedesmus vacuolatus*** | **Chlorophyta** | **Reproduction (24h)** | **Junghans et al 2003 [37]** |

* MDR-value read off from figure

**Table S2. Metal mitures.** Antagonistic and concentration additive mixtures of metal ions from Vijvers et al (2011) [38] and Xu et al (2011) [39] from which MDR-values could be calculated, sorted with the binary mixtures first and then with increasing MDR. Below are the four synergistic mixtures found of which one mixture, given in bold, was obtained from Vijvers et al (2011). The table includes information on the test species, its phylum, sub-phylum or class, the endpoint tested and the reference of the original study. The last three entries are the three extra synergistic mixtures found by the additional database study.

| **Metal 1** | **Metal 2** | **Metal 3** | **Metal 4** | **MDR** | **Test organism** | **Phylum/Subphylum or Class** | **Endpoint** | **Reference** |
| --- | --- | --- | --- | --- | --- | --- | --- | --- |
| Cd | Zn |  |  | 0.14 | *Folsomia candida* | Arthropodae/Insecta | Growth | Gestel & Hensbergen, 1997 [40] |
| Zn | Cd |  |  | 0.45 | *Strongylocentrotus intermedius* | Echinodermata | Embryogenesis | Xu et al, 2011[39] |
| Cd | Zn |  |  | 0.77 | *Chlorella sp.* | Chlorophyta | Growth | Franklin et al., 2002 [41] |
| Cd | Zn |  |  | 0.80 | *Enchytraeus albidus* | Annelidae | Reproduction | Lock & Janssen, 2002 [42] |
| Cd | Cu |  |  | 0.83 | *Chlorella sp.* | Chlorophyta | Growth | Franklin et al., 2002 [41] |
| Cu | Pb |  |  | 0.83 | *Strongylocentrotus intermedius* | Echinodermata | Embryogenesis | Xu et al, 2011[39] |
| Cu | Zn |  |  | 0.92 | *Enchytraeus crypticus* | Annelidae | Reproduction | Posthuma et al., 1997 [43] |
| Cd | Zn |  |  | <1 | *Daphnia magna* | Arthropodae/Crustacea | Mortality | Attar & Maly, 1982 [44] |
| Cd | Pb |  |  | <1 | *Enchytraeus albidus* | Annelidae | Reproduction | Lock & Janssen, 2002 [42] |
| Cu | Pb |  |  | <1 | *Enchytraeus albidus* | Annelidae | Reproduction | Lock & Janssen, 2002 [42] |
| Pb | Zn |  |  | <1 | *Enchytraeus albidus* | Annelidae | Reproduction | Lock & Janssen, 2002 [42] |
| Cu | Cd |  |  | 1 | *Strongylocentrotus intermedius* | Echinodermata | Embryogenesis | Xu et al, 2011 [39] |
| Cd | Cu |  |  | ~1 | *Enchytraeus albidus* | Annelidae | Reproduction | Lock & Janssen, 2002 [42] |
| Cu | Zn |  |  | ~1 | *Enchytraeus albidus* | Annelidae | Reproduction | Lock & Janssen, 2002 [42] |
| Cu | Zn |  |  | 1.11 | *Strongylocentrotus intermedius* | Echinodermata | Embryogenesis | Xu et al, 2011 [39] |
| Pb | Cd |  |  | 1.25 | *Strongylocentrotus intermedius* | Echinodermata | Embryogenesis | Xu et al, 2011 [39] |
| Pb | Zn |  |  | 1.42 | *Strongylocentrotus intermedius* | Echinodermata | Embryogenesis | Xu et al, 2011 [39] |
| Cu | Zn |  |  | 1.47 | *Chlorella sp.* | Chlorophyta | Growth | Franklin et al., 2002 [41] |
| Cd | Zn |  |  | 1.79 | *Folsomia candida* | Arthropodae/Insecta | Reproduction | Gestel & Hensbergen, 1997 [40] |
|  | | | |  |  |  |  |  |
|  | | | |  |  |  |  |  |
| **Mixtures including more than two metals** | | | |  |  |  |  |  |
| Cd | Cu | Zn |  | 0.71 | *Chlorella sp.* | Chlorophyta | Growth | Franklin et al., 2002 [41] |
| Cd | Cu | Zn |  | 0.71 | *Enchytraeus albidus* | Annelidae | Reproduction | Khalil et al., 1996 [45] |
| Cd | Cu | Zn | Pb | <1 | *Enchytraeus albidus* | Annelidae | Reproduction | Lock & Janssen, 2002 [42] |
| Cu | Pb | Cd |  | 1 | *Strongylocentrotus intermedius* | Echinodermata | Embryogenesis | Xu et al, 2011 [39] |
| Cu | Pb | Zn | Cd | 1 | *Strongylocentrotus intermedius* | Echinodermata | Embryogenesis | Xu et al, 2011 [39] |
| Pb | Zn | Cd |  | 1 | *Strongylocentrotus intermedius* | Echinodermata | Embryogenesis | Xu et al, 2011 [39] |
| Cu | Zn | Cd |  | 1.11 | *Strongylocentrotus intermedius* | Echinodermata | Embryogenesis | Xu et al, 2011 [39] |
| Cu | Pb | Zn |  | 1.42 | *Strongylocentrotus intermedius* | Echinodermata | Embryogenesis | Xu et al, 2011 [39] |
|  |  |  |  |  |  |  |  |  |
| **Synergistic mixtures** | | | | | |  |  |  |
| Cu | Zn |  |  | ~2 | *Gobiocypris rarus* | Cordata/Osteichthyes | Mortality (24h) | Zhu et al 2011 [46] |
| Cu | Cd |  |  | ~2 | *Gobiocypris rarus* | Cordata/Osteichthyes | Mortality (24h) | Zhu et al 2011 [46] |
| **Cd** | **Zn** |  |  | **3.57** | ***Penaeus setiferus*** | **Arthropodae/Crustacea** | **Mortality** | **Vanegas et al., 1996 [47]** |
| Cd | As |  |  | ~4 | *Daphnia magna* | Arthropodae/Crustacea | Mortality (24h) | Fikidiciki et al 2012 [48] |

**Table S3. Mixtures of antifoulants.** All mixtures of antifoulants (Antif) from which MDR-values could be calculated, sorted with the binary mixtures first and then with increasing MDR. The table includes information on the test species, its phylum, sub-phylum or class, the endpoint tested and the reference of the original study. For full chemical names and chemical class and mode of action of the antifaulants, please consult Table 1 in the manuscript. The following names are abbreviated: Irgarol1051 (Irgarol), Seanine211 (Seanine), Chlorothalonil (Chlorot.), Dichlofluanid (Dichlo), Tolylluanid (Tolyl).

| **Antif 1** | **Antif 2** | **Antif 3** | **Antif 4** | **MDR** | **Test organism** | **Phylum/Subphylum or Class** | | | | **Endpoint** | **Reference** | |
| --- | --- | --- | --- | --- | --- | --- | --- | --- | --- | --- | --- | --- |
| CuPT | Ziram |  |  | 0.02 | *Vibrio Fischeri* | | Protobacteria | | Luminescence (30min) | | | Zhou et al 2006 [49] |
| Irgarol | Seanine |  |  | 0.06 | *Vibrio Fischeri* | | Protobacteria | | Luminescence (15min) | | | Fernandez-Alba et al 2002 [50] |
| Irgarol | Chlorot. | |  | 0.15 | *Daphnia magna* | | | Arthropodae/Crustacea | Mortality (48h) | | | Fernandez-Alba et al 2002 [50] |
| Chlorot. | CuPT |  |  | 0.24 | *Artemia salina* | | | Arthropodae/Crustacea | Mortality (24h) | | | Koutsaftis and Aoyama 2007 [51] |
| Diuron | Chlorot. | |  | 0.39 | *Artemia salina* | | | Arthropodae/Crustacea | Mortality (24h) | | | Koutsaftis and Aoyama 2007 [51] |
| ZnPT | Zn |  |  | 0.40 | *Chaetoceros gracilis* | | | Chlorophyta | Growth (72h) | | | Koutsaftis and Aoyama 2006 [52] |
| Irgarol | Seanine |  |  | 0.42 | *Daphnia magna* | | | Arthropodae/Crustacea | Mortality (48h) | | | Fernandez-Alba et al 2002 [50] |
| Irgarol | 2.4-D |  |  | 0.45 | *Dunaliella tertiolecta* | | | Chlorophyta | Growth (96h) | | | Delorenzo & Serrano 2006 [53] |
| Irgarol | Dichlo. | |  | 0.46 | *Vibrio Fischeri* | | | Protobacteria | Luminescence (15min) | | | Fernandez-Alba et al 2002 [50] |
| ZnPT | Cu |  |  | 0.49 | *Chaetoceros gracilis* | | | Chlorophyta | Growth (72h) | | | Koutsaftis and Aoyama 2006 [52] |
| TBT | Seanine |  |  | 0.50 | *Scenedesmus vacuolatus* | | | Chlorophyta | Reproduction (24h) | | | Arrhenius et al 2006 [54] |
| Irgarol | Seanine |  |  | 0.50 | *Scenedesmus vacuolatus* | | | Chlorophyta | Reproduction (24h) | | | Arrhenius et al 2006 [54] |
| Tolyl. | Seanine | |  | 0.53 | *Strongylocentrotus intermedius* | | | Echinodermata | Embryogenesis | | | Wang et al 2011 [55] |
| Chlorot. | ZnPT |  |  | 0.54 | *Artemia salina* | | | Arthropodae/Crustacea | Mortality (24h) | | | Koutsaftis and Aoyama 2007 [51] |
| TBT | Irgarol | |  | 0.55 | *Scenedesmus vacuolatus* | | | Chlorophyta | Reproduction (24h) | | | Arrhenius et al 2006 [54] |
| ZnPT | Chlorot. | |  | 0.60 | *Paracentrotus lividus* | | | Echinodermata | Embryo development (48h) | | | Bellas 2008 [56] |
| ZnPT | Chlorot. | |  | 0.60 | *Paracentrotus lividus* | | | Echinodermata | Larval Growth (48h) | | | Bellas 2008 [56] |
| ZnPT | Seanine |  |  | 0.61 | *Paracentrotus lividus* | | | Echinodermata | Embryo development (48h) | | | Bellas 2008 [56] |
| Ziram | PTPB |  |  | 0.67 | *Vibrio Fischeri* | | | Protobacteria | Luminescence (30min) | | | Zhou et al 2006 [49] |
| CuPT | PTPB |  |  | 0.67 | *Vibrio Fischeri* | | | Protobacteria | Luminescence (30min) | | | Zhou et al 2006 [49] |
| CuPT | Diuron |  |  | 0.68 | *Vibrio Fischeri* | | | Protobacteria | Luminescence (30min) | | | Zhou et al 2006 [49] |
| PTPB | Diuron |  |  | 0.71 | *Vibrio Fischeri* | | | Protobacteria | Luminescence (30min) | | | Zhou et al 2006 [49] |
| Ziram | Diuron |  |  | 0.71 | *Vibrio Fischeri* | | | Protobacteria | Luminescence (30min) | | | Zhou et al 2006 [49] |
| CuPT | Seanine | |  | 0.71 | *Vibrio Fischeri* | | | Protobacteria | Luminescence (30min) | | | Zhou et al 2006 [49] |
| Seanine | DICHLO |  |  | 0.72 | *Vibrio Fischeri* | | | Protobacteria | Luminescence (30min) | | | Zhou et al 2006 [49] |
| IPBC | DICHLO |  |  | 0.72 | *Vibrio Fischeri* | | | Protobacteria | Luminescence (30min) | | | Zhou et al 2006 [49] |
| CuSO4 | IPBC |  |  | 0.72 | *Vibrio Fischeri* | | | Protobacteria | Luminescence (30min) | | | Zhou et al 2006 [49] |
| Chlorot. | Seanine |  |  | 0.73 | *Paracentrotus lividus* | | | Echinodermata | Embryo development (48h) | | | Bellas 2008 [56] |
| Seanine | Irgarol | |  | 0.73 | *Vibrio Fischeri* | | | Protobacteria | Luminescence (30min) | | | Zhou et al 2006 [49] |
| Ziram | Seanina |  |  | 0.73 | *Vibrio Fischeri* | | | Protobacteria | Luminescence (30min) | | | Zhou et al 2006 [49] |
| Ziram | Irgarol | |  | 0.74 | *Vibrio Fischeri* | | | Protobacteria | Luminescence (30min) | | | Zhou et al 2006 [49] |
| Ziram | DICHLO |  |  | 0.75 | *Vibrio Fischeri* | | | Protobacteria | Luminescence (30min) | | | Zhou et al 2006 [49] |
| ZnPT | PTPB |  |  | 0.75 | *Vibrio Fischeri* | | | Protobacteria | Luminescence (30min) | | | Zhou et al 2006 [49] |
| ZnPT | CuPT |  |  | 0.75 | *Vibrio Fischeri* | | | Protobacteria | Luminescence (30min) | | | Zhou et al 2006 [49] |
| Chlorot. | Seanine |  |  | 0.76 | *Paracentrotus lividus* | | | Echinodermata | Larval Growth (48h) | | | Bellas 2008 [56] |
| Irgarol | Seanine |  |  | 0.76 | *Selenastrum capricornutum* | | | Chlorophyta | Growth (72h) | | | Fernandez-Alba et al 2002 [50] |
| Diuron | DICHLO |  |  | 0.77 | *Vibrio Fischeri* | | | Protobacteria | Luminescence (30min) | | | Zhou et al 2006 [49] |
| ZnPT | Seanine |  |  | 0.81 | *Vibrio Fischeri* | | | Protobacteria | Luminescence (30min) | | | Zhou et al 2006 [49] |
| CuPT | DICHLO |  |  | 0.82 | *Vibrio Fischeri* | | | Protobacteria | Luminescence (30min) | | | Zhou et al 2006 [49] |
| ZnPT | DICHLO |  |  | 0.82 | *Vibrio Fischeri* | | | Protobacteria | Luminescence (30min) | | | Zhou et al 2006 [49] |
| CuPT | Irgarol | |  | 0.83 | *Vibrio Fischeri* | | | Protobacteria | Luminescence (30min) | | | Zhou et al 2006 [49] |
| Seanine | Diuron |  |  | 0.83 | *Vibrio Fischeri* | | | Protobacteria | Luminescence (30min) | | | Zhou et al 2006 [49] |
| ZnPT | Diuron |  |  | 0.84 | *Vibrio Fischeri* | | | Protobacteria | Luminescence (30min) | | | Zhou et al 2006 [49] |
| ZnPT | IPBC |  |  | 0.84 | *Vibrio Fischeri* | | | Protobacteria | Luminescence (30min) | | | Zhou et al 2006 [49] |
| PTPB | DICHLO |  |  | 0.85 | *Vibrio Fischeri* | | | Protobacteria | Luminescence (30min) | | | Zhou et al 2006 [49] |
| ZnPT | Irgarol | |  | 0.85 | *Vibrio Fischeri* | | | Protobacteria | Luminescence (30min) | | | Zhou et al 2006 [49] |
| Seanine | PTPB |  |  | 0.86 | *Vibrio Fischeri* | | | Protobacteria | Luminescence (30min) | | | Zhou et al 2006 [49] |
| CuPT | IPBC |  |  | 0.89 | *Vibrio Fischeri* | | | Protobacteria | Luminescence (30min) | | | Zhou et al 2006 [49] |
| Irgarol | Zn |  |  | 0.89 | *Chaetoceros gracilis* | | | Chlorophyta | Growth (72h) | | | Koutsaftis and Aoyama 2006 [52] |
| Ziram | IPBC |  |  | 0.89 | *Vibrio Fischeri* | | | Protobacteria | Luminescence (30min) | | | Zhou et al 2006 [49] |
| Irgarol | Dichlo. | |  | 0.90 | *Selenastrum capricornutum* | | | Chlorophyta | Growth (72h) | | | Fernandez-Alba et al 2002 [50] |
| CuSO4 | PTPB |  |  | 0.91 | *Vibrio Fischeri* | | | Protobacteria | Luminescence (30min) | | | Zhou et al 2006 [49] |
| CuSO4 | DICHLO |  |  | 0.91 | *Vibrio Fischeri* | | | Protobacteria | Luminescence (30min) | | | Zhou et al 2006 [49] |
| IPBC | Diuron |  |  | 0.98 | *Vibrio Fischeri* | | | Protobacteria | Luminescence (30min) | | | Zhou et al 2006 [49] |
| PTPB | IPBC |  |  | 0.98 | *Vibrio Fischeri* | | | Protobacteria | Luminescence (30min) | | | Zhou et al 2006 [49] |
| ZnPT | Seanine |  |  | 0.99 | *Paracentrotus lividus* | | | Echinodermata | Larval Growth (48h) | | | Bellas 2008 [56] |
| CuPT | Diuron |  |  | 0.99 | *Artemia salina* | | | Arthropodae/Crustacea | Mortality (24h) | | | Koutsaftis and Aoyama 2007 [51] |
| Cu | Irgarol | |  | 1.00 | *Tigriopus japonicus* | | | Arthropodae/Crustacea | Mortality (96h) | | | Bao et al, 2013 [57] |
| Chlorot. | Seanine |  |  | 1.00 | *Botryllus schlosseri* | | | Cordata/Tunicata | Haemocyte endpoints (cell-spreading, cytochrome-c-oxidase and GSH index) | | | Cima et al 2008 [58] |
| Irgarol | Diuron |  |  | 1.00 | *Zostera marina* | | | Plantae/angiosperm | Growth (10d) | | | Chesworth et al 2004 [59] |
| Irgarol | Atrazin |  |  | 1.00 | *Dunaliella tertiolecta* | | | Chlorophyta | Growth (96h) | | | Delorenzo & Serrano 2006 [53] |
| Seanine | IPBC |  |  | 1.02 | *Vibrio Fischeri* | | | Protobacteria | Luminescence (30min) | | | Zhou et al 2006 [49] |
| DICHLO | Irgarol | |  | 1.09 | *Vibrio Fischeri* | | | Protobacteria | Luminescence (30min) | | | Zhou et al 2006 [49] |
| Diuron | Zn |  |  | 1.14 | *Chaetoceros gracilis* | | | Chlorophyta | Growth (72h) | | | Koutsaftis and Aoyama 2006 [52] |
| CuSO4 | Diuron |  |  | 1.14 | *Vibrio Fischeri* | | | Protobacteria | Luminescence (30min) | | | Zhou et al 2006 [49] |
| CuSO4 | Seanine |  |  | 1.15 | *Vibrio Fischeri* | | | Protobacteria | Luminescence (30min) | | | Zhou et al 2006 [49] |
| CuSO4 | Irgarol | |  | 1.17 | *Vibrio Fischeri* | | | Protobacteria | Luminescence (30min) | | | Zhou et al 2006 [49] |
| ZnPT | Ziram |  |  | 1.17 | *Vibrio Fischeri* | | | Protobacteria | Luminescence (30min) | | | Zhou et al 2006 [49] |
| Cu | ZnPT |  |  | 1.23 | *Hydroides elegans* | | | Annelida/polycaeta | Mortality (48h) | | | Bao et al 2008 [60] |
| Irgarol | Cu |  |  | 1.25 | *Chaetoceros gracilis* | | | Chlorophyta | Growth (72h) | | | Koutsaftis and Aoyama 2006 [52] |
| Diuron | ZnPT |  |  | 1.25 | *Artemia salina* | | | Arthropodae/Crustacea | Mortality (24h) | | | Koutsaftis and Aoyama 2007 [51] |
| ZnPT | Cd |  |  | 1.39 | *Chaetoceros gracilis* | | | Chlorophyta | Growth (72h) | | | Koutsaftis and Aoyama 2006 [52] |
| Irgarol | Chlorot. | |  | 1.51 | *Dunaliella tertiolecta* | | | Chlorophyta | Growth (96h) | | | Delorenzon & Serrano 2006 [53] |
| Dichlo. | Seanine | |  | 1.61 | *Strongylocentrotus intermedius* | | | Echinodermata | Embryogenesis | | | Wang et al 2011 [55] |
| CuPT | Irgarol | |  | 1.61 | *Strongylocentrotus intermedius* | | | Echinodermata | Embryogenesis | | | Wang et al 2011 [55] |
| ZnPT | CuSO4 |  |  | 1.66 | *Vibrio Fischeri* | | | Protobacteria | Luminescence (30min) | | | Zhou et al 2006 [49] |
| Irgarol | TCMTB |  |  | 2.05 | *Selenastrum capricornutum* | | | Chlorophyta | Growth (72h) | | | Fernandez-Alba et al 2002 [50] |
| CuPT | CuSO4 |  |  | 2.10 | *Vibrio Fischeri* | | | Protobacteria | Luminescence (30min) | | | Zhou et al 2006 [49] |
| ZnPT | CuPT |  |  | 2.10 | *Artemia salina* | | | Arthropodae/Crustacea | Survival (24h) | | | Koutsaftis and Aoyama, 2007 [51] |
| Tolyl. | Dichlo. | |  | 2.22 | *Strongylocentrotus intermedius* | | | Echinodermata | Embryogenesis | | | Wang et al 2011 [55] |
| Diuron | ZnPT |  |  | 2.27 | *Chaetoceros gracilis* | | | Chlorophyta | Growth (72h) | | | Koutsaftis and Aoyama 2006 [52] |
| Cu | ZnPT |  |  | 2.30 | *Elasmopus rapax* | | | Arthropodae/Crustacea | Mortality (96h) | | | Bao et al 2008 [60] |
| Irgarol | ZnPT |  |  | 2.44 | *Chaetoceros gracilis* | | | Chlorophyta | Growth (72h) | | | Koutsaftis and Aoyama 2006 [52] |
| Cu | ZnPT |  |  | 2.46 | *Thalassiosira pseudonana* | | | Heterokontophyta (marine phytoplankton) | Growth (96h) | | | Bao et al 2008 [60] |
| Irgarol | Dichlo. | |  | 2.55 | *Daphnia magna* | | | Arthropodae/Crustacea | Mortality (48h) | | | Fernandez-Alba et al 2002 [50] |
| Irgarol | Seanine | |  | 2.70 | *Strongylocentrotus intermedius* | | | Echinodermata | Embryogenesis | | | Wang et al 2011[55] |
| Irgarol | Chlorot. | |  | 2.77 | *Selenastrum capricornutum* | | | Chlorophyta | Growth (72h) | | | Fernandez-Alba et al 2002 [50] |
| Irgarol | Diuron |  |  | 2.83 | *Selenastrum capricornutum* | | | Chlorophyta | Growth (72h) | | | Fernandez-Alba et al 2002 [50] |
| CuSO4 | Ziram |  |  | 2.91 | *Vibrio Fischeri* | | | Protobacteria | Luminescence (30min) | | | Zhou et al 2006 [49] |
| ZnPT | CuPT |  |  | 3.00 | *Artemia salina* | | | Arthropodae/Crustacea | Survival (24h) | | | Koutsaftis & Aoyama, 2007 [51] |
| Irgarol | Cd |  |  | 3.03 | *Chaetoceros gracilis* | | | Chlorophyta | Growth (72h) | | | Koutsaftis and Aoyama 2006 [52] |
| CuPT | Dichlo. | |  | 3.13 | *Strongylocentrotus intermedius* | | | Echinodermata | Embryogenesis | | | Wang et al 2011 [55] |
| Irgarol | Dichlo. | |  | 3.13 | *Strongylocentrotus intermedius* | | | Echinodermata | Embryogenesis | | | Wang et al 2011 [55] |
| Diuron | Cu |  |  | 4.35 | *Chaetoceros gracilis* | | | Chlorophyta | Growth (72h) | | | Koutsaftis and Aoyama 2006 [52] |
| Diuron | Cd |  |  | 5.26 | *Chaetoceros gracilis* | | | Chlorophyta | Growth (72h) | | | Koutsaftis and Aoyama 2006 [52] |
| CuPT | Seanine | |  | 5.26 | *Strongylocentrotus intermedius* | | | Echinodermata | Embryogenesis | | | Wang et al 2011 [55] |
| CuPT | Tolyl. | |  | 6.67 | *Strongylocentrotus intermedius* | | | Echinodermata | Embryogenesis | | | Wang et al 2011 [55] |
| Irgarol | Tolyl. | |  | 6.67 | *Strongylocentrotus intermedius* | | | Echinodermata | Embryogenesis | | | Wang et al 2011 [55] |
| Irgarol | TCMTB |  |  | 8.54 | *Vibrio Fischeri* | | | Protobacteria | Luminescence (15min) | | | Fernandez-Alba et al 2002 [50] |
| Irgarol | TCMTB |  |  | 9.43 | *Daphnia magna* | | | Arthropodae/Crustacea | Mortality (48h) | | | Fernandez-Alba et al 2002 [50] |
| ZnPT | CuPT |  |  | 9.63 | *Artemia salina* | | | Arthropodae/Crustacea | Mortality (24h) | | | Koutsaftis and Aoyama 2007 [51] |
| Diuron | Irgarol | |  | 10.00 | *Chaetoceros gracilis* | | | Chlorophyta | Growth (72h) | | | Koutsaftis and Aoyama 2006 [52] |
| Irgarol | Diuron |  |  | 29.04 | *Daphnia magna* | | | Arthropodae/Crustacea | Mortality (48h) | | | Fernandez-Alba et al 2002 [50] |
|  |  |  |  |  |  | | |  |  | | |  |
| **Mixtures including more than two antifoulants** | | | | |  | | |  |  | | |  |
| Irgarol | Diuron | Seanine |  | 0.28 | *Daphnia magna* | | | Arthropodae/Crustacea | Mortality (48h) | | | Fernandez-Alba et al 2002 [50] |
| Irgarol | Diuron | Seanine |  | 0.57 | *Selenastrum capricornutum* | | | Chlorophyta | Growth (72h) | | | Fernandez-Alba et al 2002 [50] |
| ZnPT | Chlorot. | Diuron |  | 0.59 | *Artemia salina* | | | Arthropodae/Crustacea | Mortality (24h) | | | Koutsaftis and Aoyama 2007 [51] |
| CuPT | Chlorot. | Diuron |  | 0.62 | *Artemia salina* | | | Arthropodae/Crustacea | Mortality (24h) | | | Koutsaftis and Aoyama 2007 [51] |
| ZnPT | Chlorot. | Seanine |  | 0.63 | *Paracentrotus lividus* | | | Echinodermata | Embryo development (48h) | | | Bellas 2008 [56] |
| ZnPT | Chlorot. | Seanine |  | 0.67 | *Paracentrotus lividus* | | | Echinodermata | Larval Growth (48h) | | | Bellas 2008 [56] |
| ZnPT | CuPT | Diuron |  | 0.92 | *Artemia salina* | | | Arthropodae/Crustacea | Mortality (24h) | | | Koutsaftis and Aoyama 2007 [51] |
| Irgarol | Diuron | Cu |  | 0.95 | *Paracentrotus lividus* | | | Echinodermata | Embryogenesis | | | Manzo et al 2008 [61] |
| ZnPT | CuPT | Chlorot. | | 1.37 | *Artemia salina* | | | Arthropodae/Crustacea | Mortality (24h) | | | Koutsaftis and Aoyama 2007 [51] |
| Irgarol | TCMTB | Dichlo. | | 2.04 | *Vibrio Fischeri* | | | Protobacteria | Luminescence (15min) | | | Fernandez-Alba et al 2002 [50] |
| Irgarol | Tolyl. | Seanine | | 2.22 | *Strongylocentrotus intermedius* | | | Echinodermata | Embryogenesis | | | Wang et al 2011 [55] |
| CuPT | Dichlo. | Seanine | | 2.63 | *Strongylocentrotus intermedius* | | | Echinodermata | Embryogenesis | | | Wang et al 2011 [55] |
| CuPT | Tolyl. | Seanine | | 2.94 | *Strongylocentrotus intermedius* | | | Echinodermata | Embryogenesis | | | Wang et al 2011 [55] |
| Irgarol | TCMTB | Dichlo. | | 3.11 | *Selenastrum capricornutum* | | | Chlorophyta | Growth (72h) | | | Fernandez-Alba et al 2002 [50] |
| CuPT | Irgarol | Seanine | | 3.45 | *Strongylocentrotus intermedius* | | | Echinodermata | Embryogenesis | | | Wang et al 2011 [55] |
| Irgarol | TCMTB | Dichlo. | | 3.73 | *Daphnia magna* | | | Arthropodae/Crustacea | Mortality (48h) | | | Fernandez-Alba et al 2002 [50] |
| Irgarol | Dichlo. | Seanine | | 4.35 | *Strongylocentrotus intermedius* | | | Echinodermata | Embryogenesis | | | Wang et al 2011[55] |
| CuPT | Irgarol | Tolyl. | | 6.67 | *Strongylocentrotus intermedius* | | | Echinodermata | Embryogenesis | | | Wang et al 2011 [55] |
| Tolyl. | Dichlo. | Seanine | | 10.00 | *Strongylocentrotus intermedius* | | | Echinodermata | Embryogenesis | | | Wang et al 2011 [55] |
| CuPT | Tolyl. | Dichlo. | | 14.29 | *Strongylocentrotus intermedius* | | | Echinodermata | Embryogenesis | | | Wang et al 2011 [55] |
| CuPT | Irgarol | Dichlo. | | 33.33 | *Strongylocentrotus intermedius* | | | Echinodermata | Embryogenesis | | | Wang et al 2011 [55] |
| Irgarol | Tolyl. | Dichlo. | | 33.33 | *Strongylocentrotus intermedius* | | | Echinodermata | Embryogenesis | | | Wang et al 2011 [55] |
| Irgarol | TCMTB | Dichlo. | Seanine | 0.09 | *Selenastrum capricornutum* | | | Chlorophyta | Growth (72h) | | | Fernandez-Alba et al 2002 [50] |
| Seanine | TBT | Irgarol | | 0.45 | *Scenedesmus vacuolatus* | | | Chlorophyta | Reproduction (24h) | | | Arrhenius et al 2006 [54] |
| Irgarol | TCMTB | Dichlo. | Seanine | 0.45 | *Daphnia magna* | | | Arthropodae/Crustacea | Mortality (48h) | | | Fernandez-Alba et al 2002 [50] |
| Irgarol | TCMTB | Dichlo. | Seanine | 0.76 | *Vibrio Fischeri* | | | Protobacteria | Luminescence (15min) | | | Fernandez-Alba et al 2002 [50] |
| ZnPT | CuPT | Chlorot. | Diuron | 2.13 | *Artemia salina* | | | Arthropodae/Crustacea | Mortality (24h) | | | Koutsaftis and Aoyama 2007 [51] |
| CuPT | Tolyl. | Dichlo. | Seanine | 3.45 | *Strongylocentrotus intermedius* | | | Echinodermata | Embryogenesis | | | Wang et al 2011 [55] |
| CuPT | Irgarol | Tolyl. | Dichlo. | 5.00 | *Strongylocentrotus intermedius* | | | Echinodermata | Embryogenesis | | | Wang et al 2011 [55] |
| CuPT | Irgarol | Tolyl. | Seanine | 5.88 | *Strongylocentrotus intermedius* | | | Echinodermata | Embryogenesis | | | Wang et al 2011 [55] |
| Irgarol | Tolyl. | Dichlo. | Seanine | 6.25 | *Strongylocentrotus intermedius* | | | Echinodermata | Embryogenesis | | | Wang et al 2011 [55] |
| CuPT | Irgarol | Dichlo. | Seanine | 7.69 | *Strongylocentrotus intermedius* | | | Echinodermata | Embryogenesis | | | Wang et al 2011 [55] |
| CuPT | Irgarol | Tolyl. | Seanine | 10.00 | *Strongylocentrotus intermedius* | | | Echinodermata | Embryogenesis | | | Wang et al 2011 [55] |

**Table S4. Additional synergistic mixtures.** Synergistic mixtures between metals and organic compounds which did not fit into any of the three categories; pesticides, metals or antifoulants, sorted with increasing MDR. The table includes information on the test species, its phylum, sub-phylum or class, the endpoint tested and the reference of the original study.

| **Metal** | **Organic chem.** | **MDR** | **Test organism** | **Phylum/Subphylum or Class** | **Endpoint** | **MDR** |
| --- | --- | --- | --- | --- | --- | --- |
| As | Malathion | 1.45 | *Tigriopus brevicornis* | Arthropodae/Crustacea | Lethality (96h) | Forget *et al.* 1999 [34] |
| Cu | Carbofuran | 1.75 | *Tigriopus brevicornis* | Arthropodae/Crustacea | Lethality (96h) | Forget *et al.* 1999 [34] |
| As | Dichlorvos | 1.96 | *Tigriopus brevicornis* | Arthropodae/Crustacea | Lethality (96h) | Forget *et al.* 1999 [34] |
| As | Carbofuran | 2.27 | *Tigriopus brevicornis* | Arthropodae/Crustacea | Lethality (96h) | Forget *et al.* 1999 [34] |
| Cd | Carbofuran | 2.27 | *Tigriopus brevicornis* | Arthropodae/Crustacea | Lethality (96h) | Forget *et al.* 1999 [34] |
| Cu | Chlorpyriphos | 2.30 | *Ceaenorhabditis elegans* | Nematoda/Chromadorae | Reproduction (96h) | Sejerøe 2011 [21] |
| Cd | Dimethoate | 2.90 | *Folsomia candida* | Arthropodae/Insecta | Reproduction (28d) | Amorim et al 2012 [4] |
| Cu | Chlorpyriphos | 4.30 | *Ceaenorhabditis elegans* | Nematoda/Chromadorae | Reproduction (96h) | Sejerøe 2011 [21] |
| Cu | Penconazole | 8.30 | *Ceaenorhabditis elegans* | Nematoda/Chromadorae | Reproduction (96h) | Sejerøe 2011 [21] |
| Cd | Dichlorvos | 8.33 | *Tigriopus brevicornis* | Arthropodae/Crustacea | Lethality (96h) | Forget *et al.* 1999 [34] |
| Cu | Penconazole | 9.40 | *Ceaenorhabditis elegans* | Nematoda/Chromadorae | Reproduction (96h) | Sejerøe 2011 [21] |

**References to Supporting Information**

1. Belden JB, Gilliom RJ, Lydy MJ (2007) How well can we predict the toxicity of pesticide mixtures to aquatic life? Int Environ Assess Manage 3: 364-372.

2. Greco WR, Bravo G, Parsons JC (1995) The search for synergy: A critical review from a response surface perspektive. Pharmacol Rev 47: 332-385.

3. Leblanc HMK, Culp JM, Baird DJ, Alexander AC, Cessna AJ (2012) Single Versus Combined Lethal Effects of Three Agricultural Insecticides on Larvae of the Freshwater Insect Chironomus dilutus. Arch Environ Contam Toxicol 63: 378-390.

4. Amorim MJB, Pereira C, Menezes-Oliveira VB, Campos B, Soares AMVM, Loureiro S (2012) Assessing single and joint effects of chemicals on the survival and reproduction of Folsomia candida (Collembola) in soil. Environ Pollut 160: 145-152.

5. Cacciatore LC, Kristoff G, Guerrero NRV, Cochon AC (2012) Binary mixtures of azinphos-methyl oxon and chlorpyrifos oxon produce in vitro synergistic cholinesterase inhibition in Planorbarius corneus. Chemosphere 88: 450-458.

6. Lydy MJ, Austin KR (2004) Toxicity assessment of pesticide mixtures typical of the Sacramento-San Joaquin Delta using Chironomus tentans. Arch Environ Contam Toxicol 48: 49-55.

7. Bielza P, Espinosa PJ, Quinto V, Abellan J, Contreras J (2007) Synergism studies with binary mixtures of pyrethroid, carbamate and organophosphate insecticides on Frankliniella occidentalis (Pergande). Pest Manage Sci 63: 84-89.

8. Laetz CA, Baldwin DH, Collier TK, Hebert V, Stark JD, Scholz NL (2009) The Synergistic Toxicity of Pesticide Mixtures: Implications for Risk Assessment and the Conservation of Endangered Pacific Salmon. Environ Health Perspec 117: 348-353.

9. Berry JA, Downton JS (1982) Environmental regulation of photosynthesis. In: Govindjee, editors. Photosynthesis, development, carbon metabolism and plant productivity. New York: Academic Press. pp. 263-343.

10. Jin-Clark Y, Lydy MJ, Zhu KY (2002) Effects of atrazine and cyanazine on chlorpyrifos toxicity in Chironomus tentans (Diptera : Chironomidae). Environ Toxicol Chem 21: 598-603.

11. Schuler LJ, Trimble AJ, Belden JB, Lydy MJ (2005) Joint toxicity of triazine herbicides and organophosphate insecticides to the midge Chironomus tentans. Arch Environ Contam Toxicol 49: 173-177.

12. Nørgaard KB, Cedergreen N (2010) Pesticide cocktails can interact synergistically on aquatic crustaceans. Environ Sci Pollut Res 17: 957-967.

13. Corbel V, Chandre F, Darriet F, Lardeux F, Hougard JM (2003) Synergism between permethrin and propoxur against Culex quinquefasciatus mosquito larvae. Med Vet Entomol 17: 158-164.

14. Duan YB, Liu SM, Ge CY, Feng XJ, Chen CJ, Zhou MG (2012) In vitro inhibition of Sclerotinia sclerotiorum by mixtures of azoxystrobin, SHAM, and thiram. Pest Biochem Physiol103: 101-107.

15. Svendsen C, Siang P, Lister LJ, Rice A, Spurgeon DJ (2010) Similarity, Independence, Or Interaction for Binary Mixture Effects of Nerve Toxicants for the Nematode Caenorhabditis Elegans. Environ Toxicol Chem29: 1182-1191.

16. Cedergreen N, Christensen AM, Kamper A, Kudsk P, Matthiasen S, Streibig JC, Sørensen H (2008) A review of independent action as a reference model for binary mixtures of compounds with different molecular target sites. Environ Toxicol Chem27: 1621-1632.

17. Belden JB, Lydy MJ (2006) Joint toxicity of chlorpyrifos and esfenvalerate to fathead minnows and midge larvae. Environ Toxicol Chem25: 623-629.

18. Johnson RM, Pollock HS, Berenbaum MR (2009) Synergistic Interactions Between In-Hive Miticides in Apis mellifera. J Econom Entomol 102: 474-479.

19. Abdel-Nasser M (1991) Determination of LC50 of Pirimor and Nuvacron on Tilapia nilotica fish. Assiut Vet Med 25: 132-139.

20. Belden JB, Lydy MJ (2000) Impact of atrazine on organophosphate insecticide toxicity. Environ Toxicol Chem19: 2266-2274.

21. Sejerøe, L. H. (2011) Toxicity of ternary mixtures tested on *Cenorhabditis elegans* -predictions and modelling [dissertation]. University of Copenhagen. 79 p.

22. Pape-Lindstrom PA, Lydy MJ (1997) Synergistic toxicity of atrazine and organophosphate insecticides contravenes the response addition mixture model. Environ Toxicol Chem16: 2415-2420.

23. Mcleese DW, Metcalfe CD (1979) Toxicity of Mixtures of Phosphamidon and Methidathion to Lobsters (Homarus-Americanus). Chemosphere 8: 59-62.

24. Anderson TD, Lydy MJ (2002) Increased toxicity to invertebrates associated with a mixture of atrazine and organophosphate insecticides. Environ Toxicol Chem21: 1507-1514.

25. Rao KRSS, Rao KSP, Sahib IKA, Rao KVR (1985) Combined Action of Carbaryl and Phenthoate on A Fresh-Water Fish (Channa-Punctatus Bloch). Ecotoxicol Environ Saf 10: 209-217.

26. Faust M, Altenburger R, Boedeker W, Grimme LH (1994) Algal toxicity of binary combinations of pesticides. Bull Environ Contam Toxicol 53: 134-141.

27. Woods M, Kumar A, Correll R (2002) Acute toxicity of mixtures of chlorpyrifos, profenofos, and endosulfan to Ceriodaphnia dubia. Bull Environ Contam Toxicol 68: 801-808.

28. Lydy MJ, Linck SL (2003) Assessing the impact of triazine herbicides on organophosphate insecticide toxicity to the earthworm Eisenia fetida. Arch Environ Contam Toxicol 45: 343-349.

29. Fulton MH, Scott GI (1991) The Effect of Certain Intrinsic and Extrinsic Variables on the Acute Toxicity of Selected Organophosphorus Insecticides to the Mummichog, Fundulus-Heteroclitus. Journal of Environmental Science and Health Part B-Pesticides Food Contam Agri Wastes 26: 459-478.

30. Pilling ED, Jepson PC (1993) Synergism Between EBI Fungicides and A Pyrethroid Insecticide in the Honeybee (Apis-Mellifera). Pest Sci 39: 293-297.

31. Marking LL, Dawson VK (1975) Method for Assessment of Toxicity Or Efficacy of Mixtures of Chemicals. U S Fish and Wildlife Service Investigations in Fish Control 67: 1-8.

32. Durairaj S, Selvarajan VR (1995) Synergistic Action of Organophosphorus Pesticides on Fish, Oreochromis-Mossambicus. J Environ Biol 16: 51-53.

33. Sahay N, Agarwal RA (1997) MGK-264-pyrethroid synergism against Lymnaea acuminata. Chemosphere 35: 1011-1021.

34. Forget J, Pavillon JF, Beliaeff B, Bocquene G (1999) Joint action of pollutant combinations (pesticides and metals) on survival (LC50 values) and acetylcholinesterase activity of Tigriopus brevicornis (Copepoda, Harpacticoida). Environ Toxicol Chem18: 912-918.

35. Moser VC, Casey M, Hamm A, Carter WH, Simmons JE, Gennings C (2005) Neurotoxicological and statistical analyses of a mixture of five organophosphorus pesticides using a ray design. Toxicol Sci 86: 101-115.

36. Moser VC, Simmons JE, Gennings C (2006) Neurotoxicological interactions of a five-pesticide mixture in preweanling rats. Toxicol Sci 92: 235-245.

37. Junghans M, Backhaus T, Faust M, Scholze M, Grimme LH (2003) Predictability of combined effects of eight chloroacetanilide herbicides on algal reproduction. Pest Manage Sci 59: 1101-1110.

38. Vijver MG, Elliott EG, Peijnenburg WJGM, De Snoo GR (2011) Response Predictions for Organisms Water-Exposed to Metal Mixtures: A Meta-Analysis. Environ Toxicol Chem30: 1482-1487.

39. Xu X, Li Y, Wang YA, Wang YH (2011) Assessment of toxic interactions of heavy metals in multi-component mixtures using sea urchin embryo-larval bioassay. Toxicol in Vitro 25: 294-300.

40. Van Gestel CA, Hensbergen PJ (1997) Interaction of Cd and Zn toxicity for Folsomia candida Willem (Collembola: Isotomidae) in relation to bioavailability in soil. Environ Toxicol Chem16: 1177-1186.

41. Franklin NM, Stauber JL, Lim RP, Petocz P (2002) Toxicity of metal mixtures to a tropical freshwater alga (Chlorella sp): The effect of interactions between copper, cadmium, and zinc on metal cell binding and uptake. Environ Toxicol Chem21: 2412-2422.

42. Lock K, Janssen CR (2002) Mixture toxicity of zinc, cadmium, copper, and lead to the potworm Enchytraeus albidus. Ecotoxicol Environ Saf 52: 1-7.

43. Posthuma L, Baerselman R, Van Veen RPM, Dirven-Van Breemen EM (1997) Single and joint toxic effects of copper and zinc on reproduction of Enchytraeus crypticus in relation to sorption of metals in soils. Ecotoxicol Environ Saf 38: 108-121.

44. Attar EN, Maly EJ (1982) Acute Toxicity of Cadmium, Zinc, and Cadmium-Zinc Mixtures to Daphnia-Magna. Arch Environ Contam Toxicol 11: 291-296.

45. Khalil MA, AbdelLateif HM, Bayoumi BM, Vanstraalen NM, VanGestel CAM (1996) Effects of metals and metal mixtures on survival and cocoon production of the earthworm Aporrectodea caliginosa. Pedobiologia 40: 548-556.

46. Zhu B, Wu ZF, Li J, Wang GX (2011) Single and joint action toxicity of heavy metals on early developmental stages of Chinese rare minnow (Gobiocypris rarus). Ecotoxicol Environ Saf 74: 2193-2202.

47. Vanegas C, Espina S, Botello AV, Villanueva S (1997) Acute toxicity and synergism of cadmium and zinc in white shrimp, Penaeus setiferus, juveniles. Bull Environ Contam Toxicol 58: 87-92.

48. Fikirdesici S, Altindag A, Ozdemir E (2012) Investigation of acute toxicity of cadmium-arsenic mixtures to Daphnia magna with toxic units approach. Turkish Journal of Zoology 36: 543-550.

49. Zhou XJ, Okamura H, Nagata S (2006) Remarkable synergistic effects in antifouling chemicals against Vibrio fischeri in a bioluminescent assay. J Health Sci 52: 243-251.

50. Fernandez-Alba AR, Hernando MD, Piedra L, Chisti Y (2002) Toxicity evaluation of single and mixed antifouling biocides measured with acute toxicity bioassays. Anal Chim Acta 456: 303-312.

51. Koutsaftis A, Aoyama I (2007) Toxicity of four antifouling biocides and their mixtures on the brine shrimp Artemia salina. Sci Tot Environ 387: 166-174.

52. Koutsaftis A, Aoyama I (2006) The interactive effects of binary mixtures of three antifouling biocides and three heavy metals against the marine algae Chaetoceros gracilis. Environ Toxicol 21: 432-439.

53. DeLorenzo ME, Serrano L (2006) Mixture toxicity of the antifouling compound irgarol to the marine phytoplankton species Dunaliella tertiolecta. J Environ Sci Health Part B-Pest Food Contam Agri Wastes 41: 1349-1360.

54. Arrhenius A, Gronvall F, Scholze M, Backhaus T, Blanck H (2004) Predictability of the mixture toxicity of 12 similarly acting congeneric inhibitors of photosystem II in marine periphyton and epipsammon communities. Aquat Toxicol 68: 351-367.

55. Wang H, Li Y, Huang HH, Xu X, Wang YH (2011) Toxicity Evaluation of Single and Mixed Antifouling Biocides Using the Strongylocentrotus Intermedius Sea Urchin Embryo Test. Environ Toxicol Chem30: 692-703.

56. Bellas J (2008) Prediction and assessment of mixture toxicity of compounds in antifouling paints using the sea-urchin embryo-larval bioassay. Aquat Toxicol 88: 308-315.

57. Bao VWW, Leung KMY, Lui GCS, Lam MHW (2013) Acute and chronic toxicities of Irgarol alone and in combination with copper to the marine copepod Tigriopus japonicus. Chemosphere 90: 1140-1148.

58. Cima F, Bragadin M, Ballarin L (2008) Toxic effects of new antifouling compounds on tunicate haemocytes I. Sea-Nine 211 (TM) and chlorothalonil. Aquat Toxicol 86: 299-312.

59. Chesworth JC, Donkin ME, Brown MT (2004) The interactive effects of the antifouling herbiciddes Irgarol 1051 and Diuron on the seagrass *Zostera marina* (L.). Aquat Toxicol 66: 293-305.

60. Bao VWW, Leung KMY, Kwok KWH, Zhang AQ, Lui GCS (2008) Synergistic toxic effects of zinc pyrithione and copper to three marine species: Implications on setting appropriate water quality criteria. Mar Pollut Bull 57: 616-623.

61. Manzo S, Buono S, Cremisini C (2008) Predictability of copper, irgarol, and diuron combined effects on sea urchin Paracentrotus lividus. Arch Environ Contam Toxicol 54: 57-68.
